# Airway and Gut Dysbiosis, Infectious Exacerbations and Pulmonary Vascular Remodelling in COPD-Associated Pulmonary Hypertension: Microbial Mechanisms, Causal Uncertainty and Therapeutic Implications

**DOI:** 10.3390/microorganisms14091954

**Published:** 2026-09-03

**Authors:** Nilufar Akhmedova, Gulomjon Kholov, Ulugbek Ochilov, Nodira Shonazarova, Otabek Yuldashev, Gulkhayo Olimova, Sabina Istamova, Sitora Mukhammadiyeva, Nozima Kenjayeva, Jahongir Sharipov

**Affiliations:** 1 Department of Nephrology and Hemodialysis, Bukhara State Medical Institute, Bukhara 200100, Uzbekistan; nilufarakhmedova230474@gmail.com (N.A.); otabekmyuldashev@gmail.com (O.Y.); olimova.gulhayo@bsmi.uz (G.O.); istamovnasabina@gmail.com (S.I.); sitoramuhammadiyeva1125@gmail.com (S.M.); nozimakenjayeva88@gmail.com (N.K.); jahongirsharipov09@gmail.com (J.S.); 2 Interfaculty Department of Foreign Languages, Bukhara State University, Bukhara 200100, Uzbekistan; elnurbeast@gmail.com; 3 Department of Foreign Languages and Social Sciences, Asia International University, Bukhara 200100, Uzbekistan; 4 Department of Internal Diseases No. 3, Samarkand State Medical University, Samarkand 140100, Uzbekistan; drshnodira@mail.ru

**Keywords:** chronic obstructive pulmonary disease, pulmonary hypertension, microbiota, host–pathogen interactions, *Haemophilus influenzae*, disease exacerbation, endothelial dysfunction, vascular remodelling, short-chain fatty acids, gut–lung axis

## Abstract

Pulmonary hypertension complicating chronic obstructive pulmonary disease (COPD-PH) has a worse prognosis than airflow limitation alone but its pathogenesis is thought to be primarily due to alveolar hypoxia affecting pulmonary circulation. Two observations are not fully explained by this model. First, the majority of exacerbations of COPD are infectious and there is a stable frequent-exacerbator phenotype that is not fully accounted for by spirometric severity. Second, the airways of COPD are not sterile. They are home to a resident microbial community that changes as the disease progresses and the gut microbial community also undergoes disease-associated changes. This review examines whether microbial factors actively contribute to pulmonary vascular remodelling or merely reflect advanced disease. Three candidate exposures are considered: chronic airway colonisation, dysbiosis of the airway and gut communities and recurrent infectious exacerbation. We then examine the virulence mechanisms and host-recognition pathways of non-typeable Haemophilus influenzae, Streptococcus pneumoniae, Moraxella catarrhalis, Pseudomonas aeruginosa, rhinovirus, influenza viruses, respiratory syncytial virus and SARS-CoV-2. We then trace the pathways linking microbial recognition to vascular injury: Toll-like receptor signalling and NF-κB activation; interleukin-6, tumour necrosis factor-α and interleukin-1β; endothelin-1 upregulation against nitric oxide depletion; reactive oxygen species and hypoxia-inducible factor signalling converging with hypoxic pulmonary vasoconstriction; and, along the gut–lung axis, lipopolysaccharide translocation, trimethylamine N-oxide and depletion of short-chain fatty acid-producing taxa. Therapeutically, there is evidence and a vascular rationale for interventions that decrease exacerbation frequency. Pulmonary vasodilators have a sound physiological rationale, although their clinical efficacy is frequently limited by worsening ventilation–perfusion mismatch. Current evidence supports biological plausibility rather than demonstrated causality: no direct longitudinal human study has established that microbial alterations or microbial exposure cause pulmonary vascular remodelling in COPD-PH. We identify study designs capable of directly testing this hypothesis.

## 1. Introduction

Chronic obstructive pulmonary disease (COPD) is a major and increasing burden in the adult population and is one of the most common causes of death globally [1]. It is punctuated by acute exacerbations that are more than temporary interruptions of disease stability; they frequently mark periods of accelerated disease progression [2,3].

A significant minority of patients develop pulmonary hypertension, which is associated with decreased exercise capacity, increased hospitalisation and decreased survival [4,5]. The severity of its symptoms is only loosely correlated with the severity of airflow obstruction and spirometry does not adequately reflect the mechanisms driving the vascular component of the disease [5].

The traditional explanation is given by alveolar hypoxia. Chronic hypoxia causes pulmonary vasoconstriction, chronic vasoconstriction causes wall stress, and wall stress causes remodelling [6]. This sequence is well established and supported by substantial experimental and clinical evidence. However, this mechanism does not fully explain the observed haemodynamic abnormalities. Pulmonary vascular abnormalities have been shown in smokers without any lung function impairment and in patients with mild disease, prior to the onset of any significant hypoxaemia [5,7]. Endothelial dysfunction develops early in disease and the subsequent intimal changes do not correlate well with oxygen tension [7].

These observations suggest that additional biological mechanisms may contribute. This review therefore examines whether microbial processes represent one such mechanism.

Three lines of evidence provide the rationale for this hypothesis. The first is epidemiological: infection is the cause of the great majority of exacerbations and patients are reproducibly different in their rate of exacerbation—a characteristic which is stable over years and not fully explained by disease stage [2,8]. The second is microbiological: the lower airways contain a resident microbial community and as disease progresses, the diversity of the community decreases and the composition of the community changes towards *Proteobacteria* and this change is measurable not only during acute events but also during clinical stability [9,10,11]. The third is mechanistic: the signalling pathways activated by bacterial and viral recognition (Toll-like receptor (TLR) engagement, NF-κB activation, release of interleukin-6 (IL-6) and tumour necrosis factor-α (TNF-α)) are the same pathways implicated in pulmonary vascular remodelling elsewhere [12,13,14].

Each observation provides supportive evidence, although none is independently conclusive. If there were no interaction between the two, severe COPD would be expected to be associated with frequent infection and pulmonary vascular disease and the two would be expected to co-occur. Rather than postponing this discussion until the limitations section, we address it explicitly in Section 8.

The dysbiosis of the airway and gut in COPD and the changes in the microbiome in pulmonary hypertension have been extensively reviewed. But the possible interplay of these processes in the development of pulmonary vascular remodelling in COPD has been much less studied. This review brings these fields together by integrating pathogen-specific mechanisms, recurrent infectious exacerbations, airway and gut microbial ecology and pulmonary vascular biology into a unified conceptual framework.

**Figure 1:**
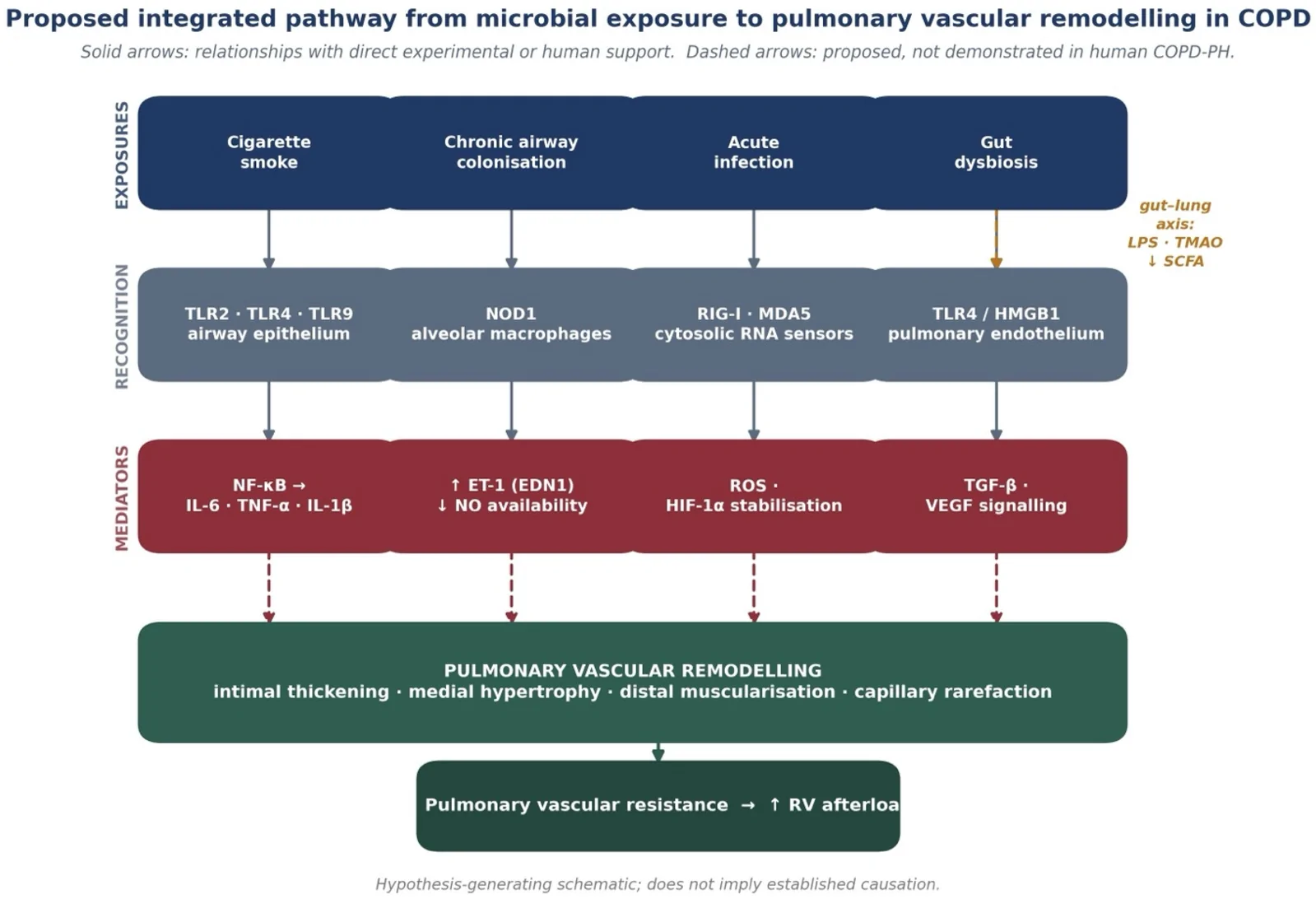
Proposed integrated pathway from microbial exposure to pulmonary vascular remodelling in COPD. Schematic representation, based on published evidence, of the routes by which microbial factors may contribute to pulmonary vascular pathology. Four tiers are shown. Exposures: cigarette smoke, chronic airway colonisation, acute infection and gut dysbiosis. Recognition: TLR2, TLR4 and TLR9, NOD1 and cytosolic RNA sensors (RIG-I, MDA5) expressed on airway epithelium, alveolar macrophages and pulmonary endothelium. Mediators: NF-κB-driven transcription of IL-6, TNF-α, IL-1β and EDN1; ET-1 elevation with reduced NO availability; ROS generation; HIF-1α stabilisation; TGF-β and VEGF signalling. Vascular outcome: intimal thickening, medial hypertrophy, distal muscularisation and capillary rarefaction, raising PVR and imposing RV afterload. Solid arrows indicate relationships supported by direct experimental or human evidence; dashed arrows indicate proposed relationships not yet demonstrated in human COPD-PH. Abbreviations as in the abbreviation list. The cascade is hypothesis-generating and does not imply established causation.

We have three goals: to compile the microbiological and mechanistic data linking infection and dysbiosis to pulmonary vascular disease; to critically evaluate whether this data provides evidence for a causal role; and to discuss the implications for therapy, distinguishing between those with proven clinical efficacy and those based on physiological rationale. An overview of the proposed mechanistic pathways discussed throughout this review is presented in Figure 1.

### Literature Search and Review Approach

This narrative review is based on a comprehensive search of PubMed, Scopus and Web of Science for English-language publications available up to 27 July 2026. Search terms included those related to chronic obstructive pulmonary disease, pulmonary hypertension, airway microbiome, gut microbiome, dysbiosis, infectious exacerbations, pulmonary vascular remodelling, endothelial dysfunction and relevant bacterial and viral pathogens. Special attention was given to current clinical guidelines, systematic reviews, randomised controlled trials, longitudinal cohort studies, experimental human infection studies and mechanistic studies that shed light on the biological pathways discussed in this review. Given the limited direct evidence of a link between microbial exposure and pulmonary vascular remodelling in COPD, findings from studies on pulmonary arterial hypertension and experimental models of pulmonary hypertension were included where they provided biologically plausible and clearly identified indirect evidence. This review was performed as a narrative synthesis of the literature available and was not conducted as a systematic review or with a formal meta-analysis.

The search was structured around four concept blocks, with synonyms combined within each block using OR and the blocks combined using AND: (i) chronic obstructive pulmonary disease OR COPD OR emphysema OR chronic bronchitis; (ii) pulmonary hypertension OR pulmonary vascular remodelling OR pulmonary vascular resistance OR endothelial dysfunction OR right ventricular function; (iii) microbiome OR microbiota OR dysbiosis OR colonisation OR bacterial load OR the individual bacterial and viral pathogen names listed above; and (iv) exacerbation OR infection OR inflammation OR Toll-like receptor OR short-chain fatty acid OR lipopolysaccharide OR trimethylamine N-oxide. Records were screened at the title and abstract level and included if they included reports of human COPD or COPD-PH populations, clinically relevant pulmonary hypertension populations from which mechanistic inference could be made or experimental studies that addressed one of the pathways discussed; publications not in English and studies that did not address either microbial exposure or pulmonary vascular biology were not used. Guideline documents, systematic reviews, randomised trials, longitudinal cohorts and experimental human infection studies were preferred to cross-sectional and single-centre reports. In keeping with the narrative design of this synthesis, no duplicate independent screening, formal risk-of-bias instrument, PRISMA flow accounting or protocol registration was used, nor is any claimed.

**Evidence grading.** The literature relevant to this hypothesis is from populations and models that vary widely in their proximity to human COPD-PH, so we used a four-tier evidence framework throughout the text and tables. Tier 1 comprises direct human COPD-PH evidence with pulmonary vascular or haemodynamic endpoints; Tier 2 comprises human COPD evidence without vascular endpoints; Tier 3 comprises evidence from pulmonary arterial hypertension or other pulmonary hypertension populations; and Tier 4 comprises animal and in vitro evidence. Direct evidence denotes studies of human COPD-PH in which pulmonary vascular or haemodynamic phenotyping was performed. Moderate (indirect clinical) evidence includes human COPD studies that are relevant to a proposed pathway but do not assess pulmonary vascular involvement and clinically relevant evidence from other pulmonary hypertension populations from which extrapolation is needed. Low or mechanistic, evidence denotes animal models, hypoxic-exposure models, pulmonary arterial hypertension models, in vitro and molecular studies and inference from general vascular biology. The labels refer to the directness of the evidence for the specific COPD-PH microbial hypothesis, NOT to the methodological quality of the underlying research: a rigorous rodent experiment is rated low because it is remote from human COPD-PH, not because it is of low methodological quality. Where the tables use the terms low, moderate and high, they refer to this framework and high is reserved for randomised or otherwise direct human evidence for the outcome named in the relevant column.

## 2. COPD-Associated Pulmonary Hypertension: Clinical Context

According to the current ESC/ERS guidelines, pulmonary hypertension is defined as a mean pulmonary arterial pressure of >20 mmHg at rest (previously >25 mmHg) and pre-capillary disease is defined as a wedge pressure of ≤15 mmHg and pulmonary vascular resistance of >2 Wood units [4]. PH complicating lung disease is Group 3. The 2022 guidelines distinguish between non-severe PH (pre-capillary haemodynamics with PVR > 2 to ≤5 WU) and severe PH (PVR > 5 WU) within Group 3 [4]. Severe PH is still a formal haemodynamic classification in the 2022 document on PH associated with lung disease and the descriptive term disproportionate pulmonary vascular involvement is complementary to, not a replacement for, the definition of severe PH (Table 1).

These observations have important clinical implications. Non-severe COPD-PH is common and acts in many respects as a marker of parenchymal disease. Severe COPD-PH is rare and patients with COPD-PH have a haemodynamic profile more similar to pulmonary arterial hypertension than to their airflow limitation. This mismatch led to the idea of a pulmonary vascular phenotype of COPD, in which vascular disease develops before the onset of airway disease [5].

Reported prevalence varies according to the population studied and the diagnostic methods used. The prognostic weight is the same: pulmonary hypertension is an independent predictor of mortality independent of FEV_1_ and vascular severity is a better predictor of outcome than obstruction [5].

Early detection remains a major clinical challenge. Exertional dyspnoea, fatigue and reduced exercise tolerance are indistinguishable from symptoms of underlying lung disease. When right ventricular dysfunction becomes apparent, remodelling is present and, at present, irreversible. Right heart catheterisation is still the gold standard but is invasive and difficult to justify if no disease-specific vascular therapy is recommended. The diagnostic performance of echocardiography is limited in COPD because hyperinflation frequently compromises acoustic windows, tricuspid regurgitant jets are often unobtainable and estimated pulmonary pressures correlate poorly with invasive measurements.

This observation is particularly relevant to the present hypothesis. If the vascular involvement starts early and is silent, then the exposures relevant to the initiation of the vascular involvement are in mild and moderate disease, not in the end-stage populations from which most pathological specimens are obtained. Explant studies have reported on advanced remodelling [15] but cannot determine what triggered it. This is also true for the microbiological literature, in which sampling is most easily done in the most severely affected patients with the highest bacterial load.

## 3. The Pulmonary Vascular Substrate

To determine if microbial factors play a role in vascular pathology, one must first define the nature of vascular pathology.

Pulmonary vascular remodelling involves structural alterations affecting all three layers of the vessel wall. The most consistent finding is intimal thickening, which consists of smooth muscle proliferation and deposition of elastic and collagen fibres; medial hypertrophy is seen in muscular arteries and distal muscularisation is the extension of smooth muscle into vessels that do not normally have it [7,16]. Venous remodelling with intimal fibrosis and arterialisation is also observed in explanted lungs of transplant recipients, which makes the conventional pre-capillary framing difficult [15]. In advanced emphysema, alveolar destruction removes capillary bed and this rarefaction adds to increased resistance in a mechanically different way: by decreasing cross-sectional area without remodelling the remaining vessels [7].

Pulmonary vascular remodelling is initiated by endothelial injury. Endothelial dysfunction has been shown throughout the spectrum of severity, in patients with end-stage disease and in patients undergoing lobectomy for other reasons [7]. It is characterised by an imbalance of vasoactive mediators, with decreased nitric oxide and prostacyclin and increased endothelin-1 (ET-1) and angiotensin II [7,17]. Of particular interest is ET-1, which acts as a vasoconstrictor and mitogen, stimulating smooth muscle proliferation as well as tone.

Remodelling at the cellular level includes the development of a proliferative and apoptosis-resistant phenotype of pulmonary artery endothelial cells, the switch from contractile to synthetic phenotype of smooth muscle cells, adventitial fibroblast differentiation and endothelial-to-mesenchymal transition of cells populating the thickened intima [12,16]. This programme is almost exclusively from pulmonary arterial hypertension and not COPD-PH and whether it is the same in the setting of less haemodynamic burden and a very different parenchymal context is assumed, not proven.

Two observations are particularly relevant to the proposed hypothesis. Vascular abnormality is not just a result of low oxygen tension—elevated circulating endothelial microparticles have been reported in smokers with normal lung function [5]. The endothelium already damaged by smoking is likely to react abnormally to further insults [7] and provides a potential mechanism by which intermittent inflammatory events may have a cumulative structural effect in a vessel bed already damaged by smoking.

## 4. Infectious Exacerbation and the Frequent-Exacerbator Phenotype

Most exacerbations of COPD are due to infection, with bacteria responsible for about half and respiratory viruses for about a third, with co-detection occurring frequently and the two types of infection interacting rather than being independent of each other [13,14]; in a hospital-based series in which viral and bacterial pathogens were sought systematically, patients with co-detection were the most likely to be readmitted [8].

The spectrum of pathogens isolated during COPD exacerbations is remarkably consistent across clinical settings. The most common bacteria isolated are *Haemophilus influenzae*, mostly non-typeable strains, *Streptococcus pneumoniae* and *Moraxella catarrhalis*, with *Pseudomonas aeruginosa* being found in more advanced disease and in the presence of bronchiectatic change [13,18]. Rhinovirus is the most common virus, followed by influenza, respiratory syncytial virus, parainfluenza and, since 2020, SARS-CoV-2 [13,14].

The ECLIPSE study fundamentally changed the current understanding of COPD exacerbations. The ECLIPSE cohort, which followed 2138 patients for three years, showed that susceptibility to exacerbation is clustered within individuals and is stable over time [2]. Importantly, susceptibility was seen in all severity strata: moderate-disease patients could exacerbate often and severe-disease patients could not. The single best predictor of future exacerbations was previous exacerbation history, not spirometry [2].

These findings provide an important framework for the causal appraisal developed in Section 8. If exacerbation frequency was only dependent on the severity of the disease, then any correlation with vascular outcome would be fully accounted for by severity. The association is not only potentially informative of something other than the extent of the disease but also the frequency varies significantly between severity strata.

The phenotype is associated with increased airway and systemic inflammation at baseline, changes in lower airway colonisation and apparent increased susceptibility to viral infection [3,19]. But frequent exacerbators also have more extrapulmonary morbidity, including cardiovascular disease [3]—a relationship that will be reiterated when we ask whether infection causes vascular disease or simply is associated with it.

Exacerbations represent major biological events rather than temporary interruptions of disease stability. They are linked to a faster rate of FEV_1_ decline and patients with more frequent events decline faster [2,3]. The physiological disturbance during an episode is itself relevant: the greater the obstruction, the greater dynamic hyperinflation; the worse is ventilation–perfusion matching, the lower the arterial oxygen tension and the worse the hypoxaemia, the greater the hypoxic pulmonary vasoconstriction across the greater proportion of lung; the greater the coexisting hypercapnia, the greater the exacerbation of that response; and the greater the systemic inflammation, the greater the increase in cardiac output. The net result is a transient but significant increase in pulmonary arterial pressure on top of any chronic increase.

Patients experiencing recurrent exacerbations over many years are exposed to repeated episodes of haemodynamic stress that may cumulatively contribute to pulmonary vascular remodelling. It is not known whether that exposure results in structural change or it resolves without residue. The argument for a cumulative effect is at least as strong as the argument for the chronic hypoxia hypothesis.

All this work is subject to a methodological caution. The majority of epidemiological studies have used treatment received (antibiotics, corticosteroids, hospitalisation) to define exacerbations, not physiological or microbiological criteria [2]. These definitions reflect healthcare utilisation as well as biology and miss unreported episodes; symptom-diary studies always find more events than prescription-record studies. This misclassification would bias toward the null and a true effect might be missed, for any analysis comparing exacerbation frequency to vascular outcome.

## 5. Pathogen-Specific Mechanisms and Microbial Virulence

The receptors they engage, the host cytokine responses they elicit and the strategies by which they persist vary from organism to organism. These differences must be considered in any account of the relationship between infection and vascular consequence and are summarised in Table 2.

### 5.1. Non-Typeable Haemophilus influenzae

The most prevalent bacterial pathogen in COPD is non-typeable *H. influenzae* (NTHi), which presents the strongest evidence for a causal role in disease progression. It is involved in about half of the exacerbations that are bacterially mediated and, importantly for the current discussion, remains in the lower airway between exacerbations [18,20].

Recognition is mainly via TLR2 and TLR4. The P6 outer-membrane lipoprotein is a TLR2 activator, while LOS is a TLR4 activator [21]. Both lead to activation of NF-κB through the MyD88 pathway, which results in the transcription of the cytokine genes *TNF*, *IL1B* and *IL6*; in bronchial epithelial infection models, NTHi also upregulates the transcription of *TLR6* and *JUN* in addition to these cytokines [22].

There are multiple adaptations that work together to ensure persistence. Antigenic escape is possible by phase variation of surface structures. Biofilm formation provides tolerance to both antibiotics and phagocytosis. Intracellular localisation in epithelial cells may limit the accessibility to some humoral immune mechanisms and is one of several factors suggested to contribute to persistence [18,20,23]. These behaviours are reproduced in in vitro models of persistent airway infection, which have become the primary experimental model for studying persistent airway infection [23].

**Figure 2:**
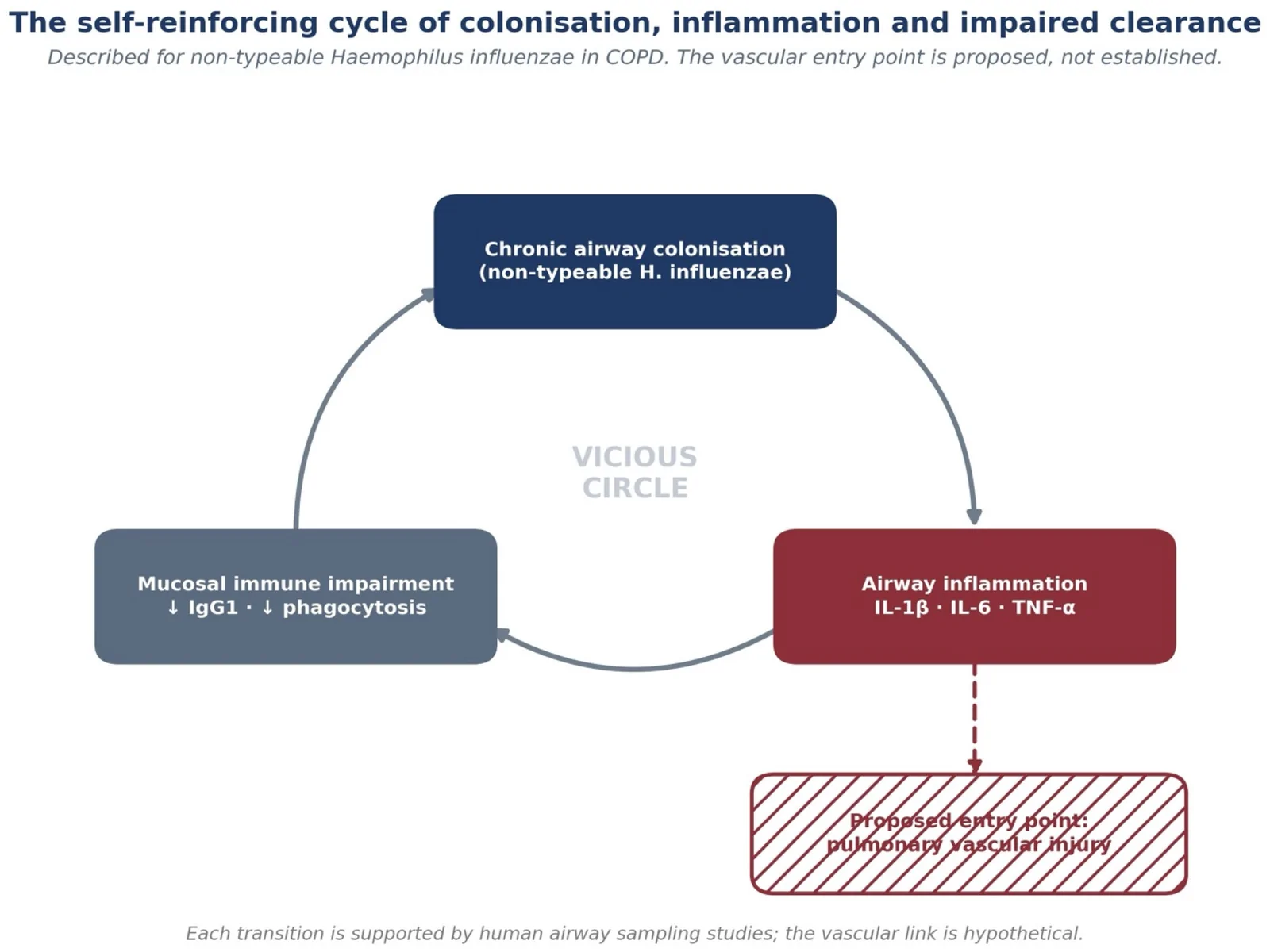
The self-reinforcing cycle of colonisation, inflammation and impaired clearance. Circular schematic illustrating the “vicious circle” described for non-typeable *Haemophilus influenzae* in COPD. Chronic colonisation drives airway inflammation; inflammation contributes to mucosal immune impairment, including compartmentalised IgG1 deficiency and reduced macrophage phagocytic capacity; impaired clearance in turn permits continued colonisation. The point at which vascular injury is proposed to enter the cycle is marked at the inflammation node. Evidence for each transition derives from human airway sampling studies; the vascular entry point is hypothetical.

This persistence is facilitated by host factors. The total and NTHi-specific IgG1 levels are decreased in bronchoalveolar lavage but not in serum of NTHi-colonised patients, suggesting a compartmentalised defect of the airways rather than a systemic immunodeficiency [24]. These same patients had significantly greater lavage IL-1β and a history of more frequent exacerbations [24]. The relationship is a self-sustaining one. Colonisation leads to inflammation, inflammation leads to impaired clearance and impaired clearance leads to further colonisation—the “vicious circle” that is characteristic of NTHi in COPD [21] (Figure 2).

Two properties of NTHi are directly relevant to the proposed vascular mechanism: it is associated with the frequent-exacerbator phenotype [24,25] and it is able to maintain airway production of IL-1β and IL-6 during clinical stability.

### 5.2. Streptococcus pneumoniae

*S. pneumoniae* is the second most commonly isolated bacterium in exacerbations [13]. Its main virulence factors include the polysaccharide capsule, which hinders phagocytosis and pneumolysin, a cholesterol-dependent cytolysin that creates pores in host membranes, activates complement and induces inflammasome-mediated IL-1β production.

Pneumolysin deserves particular attention because it directly disrupts endothelial integrity. The loss of barrier integrity is a direct effect of pore formation and the subsequent inflammasome activation enhances IL-1β signalling. In contrast to NTHi, *S. pneumoniae* is more likely to cause acute episodes of disease than chronic colonisation of the COPD airway and therefore its contribution is likely to be intermittent and intense.

### 5.3. Moraxella catarrhalis

*M. catarrhalis* is isolated in a significant minority of exacerbations and has a well-defined recognition profile. Its LOS is recognised by a CD14–TLR4 complex and whole organisms (live or heat-killed) are recognised by TLR2, TLR4 and TLR9, which signal through both MyD88- and TRIF-dependent pathways [26]. The organism is internalised by respiratory epithelial cells via a trigger-like mechanism and triggers TLR2- and partially NOD1-dependent inflammatory responses [27]. TLR2 also controls its adhesion and invasion of alveolar epithelial cells [28].

Co-infection studies are informative in terms of the microbial ecology of the airway. *M. catarrhalis* and NTHi induce overlapping but quantitatively different cytokine profiles in bronchial epithelial models, with *M. catarrhalis* inducing more IFN-α2, CCL5, CCL3, CXCL10 and CXCL11 and NTHi inducing more *TNF*, *IL1B* and *IL6* [22]. The two organisms also have differential effects on the epithelial tight junction components, with *M. catarrhalis* upregulating claudin-4, which has implications for barrier integrity and thus exposure of the vascular compartment to luminal contents [22].

### 5.4. Pseudomonas aeruginosa

*P. aeruginosa* is seen more commonly in late disease, in patients who have had multiple courses of antibiotics and in the presence of bronchiectatic change [13]. It is very persistent due to its virulence mechanisms: type III secretion system, biofilm formation, quorum sensing, and pyocyanin production. Pyocyanin is a direct source of ROS, which is a mechanism of endothelial injury not shared by the other organisms discussed here.

This organism is the most extreme example of the confounding problem discussed in Section 8.2. Patients colonised with *P. aeruginosa* have the greatest impairment in lung function, the most frequent exacerbations and the most vascular disease. Evidence of its specific association with vascular outcome should thus be interpreted with special caution.

### 5.5. Respiratory Viruses

Rhinovirus is the most commonly identified virus in exacerbations of COPD and experimental infection studies provide some of the most mechanistically informative data in this area. Experimental rhinovirus infection resulted in secondary bacterial infection in 60% of patients with moderate COPD compared to approximately 10% of smokers with normal lung function and non-smokers [13]. Rhinovirus infection also causes an increase in the existing bacterial flora, with *H. influenzae* being the most prominent—which is not seen in healthy people [13].

**Figure 3:**
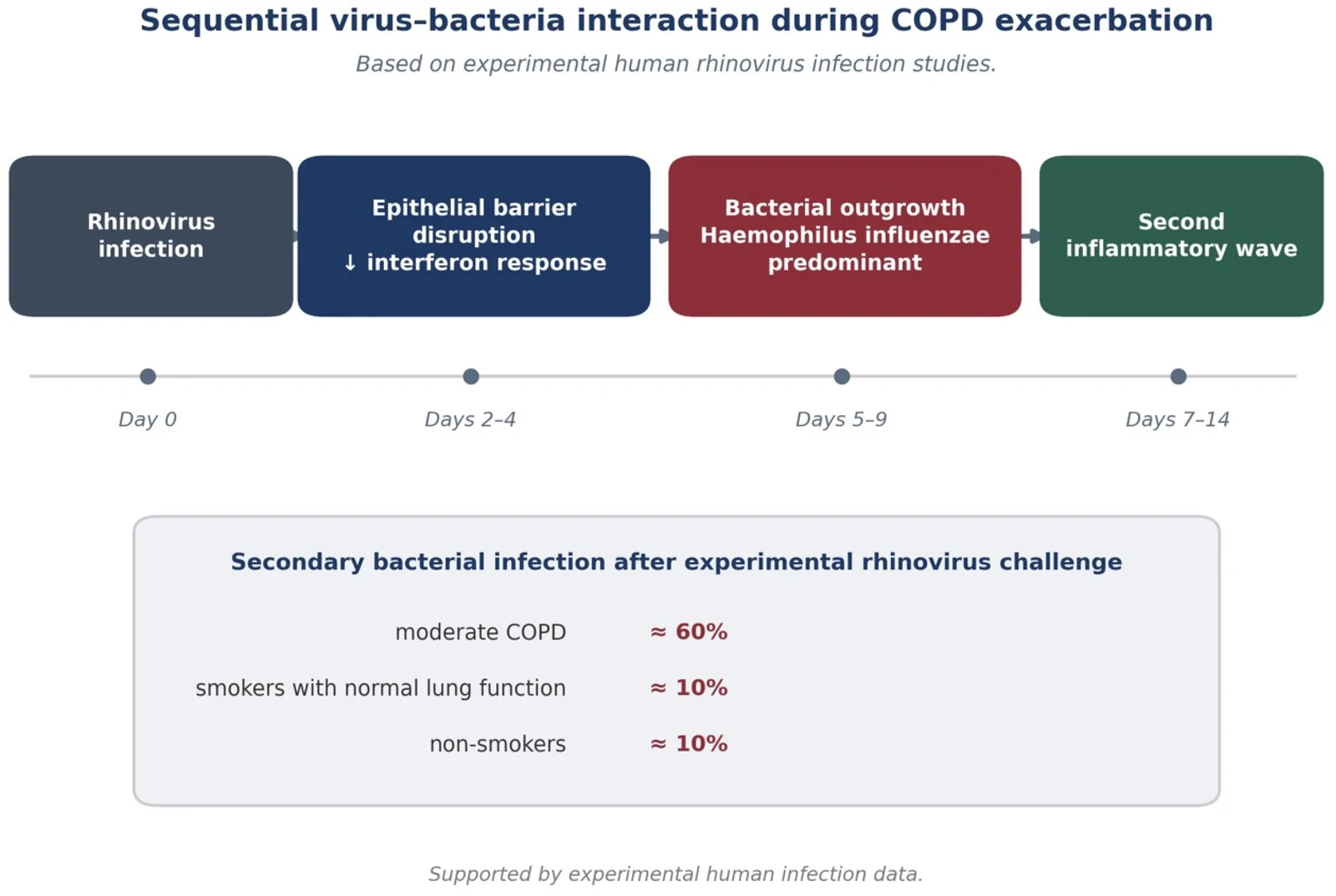
Sequential virus–bacteria interaction during COPD exacerbation. Timeline schematic based on experimental rhinovirus infection studies. Viral infection disrupts epithelial barrier function and attenuates interferon responses, permitting outgrowth of the resident bacterial community—*Haemophilus influenzae* predominating—and generating a second inflammatory wave. Annotated with reported frequencies of secondary bacterial infection following experimental rhinovirus challenge: approximately 60% in patients with moderate COPD versus approximately 10% in smokers with normal lung function and in non-smokers. This sequence is supported by experimental human infection data.

Here, the directionality is important. Viral infection causes a disruption of epithelial barrier function and antiviral defence, allowing bacterial growth and a second inflammatory wave (Figure 3). Many exacerbations are therefore thought to be due to a single organism and represent a sequential viral–bacterial insult. Differentiating between viral and bacterial exacerbations may be an artificial division that is not supported by biology.

Influenza causes greater epithelial damage and more potent systemic inflammatory responses than rhinovirus [14]. Co-infection models with NTHi reveal exacerbated inflammatory lung injury and altered gut microbiota [29]—a connection between the pathogen-specific and gut–lung axis material of this review and one of the few experimental demonstrations that respiratory infection alters intestinal communities.

Respiratory syncytial virus is now known to be present in adults with COPD and, unlike other respiratory viruses, can be detected in a proportion of patients when they are clinically stable. This suggests that chronic low-level infection may be a factor in chronic low-level inflammation but the evidence is limited and the clinical relevance is unclear.

Unlike other pathogens discussed in this article, SARS-CoV-2 is characterised by vascular manifestations. The virus enters cells via angiotensin-converting enzyme 2, which is found on respiratory epithelium and to a lesser extent on pulmonary endothelium [30]. Endothelial injury, complement activation and microvascular thrombosis are well described in severe COVID-19 [31,32]. It is unclear whether these are due to direct productive infection of pulmonary endothelial cells or indirect injury due to systemic inflammation, complement activation and hypoxaemia. In a synthesis of multi-organ involvement across 161 studies, biomarkers of endothelial dysfunction were raised in most patients and organ dysfunction persisted for months after the acute illness [32].

Whether endothelial abnormality persists in pulmonary circulation after respiratory infection, as it has been reported to do in other vascular beds, has not been studied.

It is not yet known if COVID-19 is a unique mechanism or a particularly severe example of a common mechanism. It does, however, illustrate that infection with a respiratory pathogen can be followed by persistent endothelial abnormality, which is relevant to the broader argument but is based on a virus with unusual tropism and thus may not be generalisable to the bacterial pathogens that are the major cause of COPD exacerbation.

## 6. Colonisation Versus Infection: Airway Dysbiosis and Host Immunity

This review focuses on the distinction between colonisation and infection, which is not as straightforward as it may seem.

### 6.1. The Airway Microbiome in Health and COPD

Throughout this review, dysbiosis is used in an operational sense: a reproducible alteration in the composition, diversity or functional potential of a microbial community relative to an appropriate comparator group, demonstrated in more than one dataset. It is not used as a synonym for any compositional difference, and a difference in community structure is not assumed to be pathological in itself. The lower airways are now recognised to harbour a low-biomass resident microbial community. They contain a low-biomass community, continuously seeded by microaspiration and influenced by clearance, in health dominated by *Prevotella*, *Veillonella* and *Streptococcus* [10,33,34,35,36,37].

This community is altered in COPD. Alpha diversity decreases and composition changes to *Proteobacteria*, *Haemophilus* and *Streptococcus* become more abundant [11,38,39]. Meta-analysis of oral, airway and intestinal compartments shows that the airway microbiota of stable COPD and exacerbation is enriched with *Streptococcus* and *Haemophilus* compared to healthy controls [11]. Within-individual diversity is significantly lower in the *Haemophilus*-dominated patients, both by Shannon and Simpson indices [25] (Table 3); reference [25] remains a preprint that has not completed peer review, and it is cited here as preliminary supporting evidence only.

A notable feature of these microbial alterations is their persistence during periods of clinical stability. When well, frequent exacerbators have distinguishable sputum signatures, including fewer amplicon sequence variants, lower alpha diversity and distinct beta diversity [9]. That study was a single-centre cross-sectional comparison in which the frequent-exacerbator phenotype was defined by exacerbation history over the preceding year, so these signatures are associated with a history of frequent exacerbations rather than predictive of subsequent events and airway dysbiosis has been shown to accelerate subsequent lung function decline [40]. These microbial alterations persist beyond acute exacerbations and are detectable during periods of clinical stability.

### 6.2. What Is the Difference Between Colonisation and Infection?

Conventional microbiology has a binary perspective on the presence of organisms. Microbiome data clearly indicate that this is not sufficient. The variable is community structure and the same organism can be a minor or dominant member of the community [41,42].

Sputum bacterial burden correlates with airway IL-1β [25]. This association is cross-sectional and does not prove that the amount of bacteria is responsible for the inflammatory response but it is consistent with a gradient association. This supports a continuum model, where persistent low-level colonisation may sustain chronic low-grade inflammation, with expansion events that result in clinical exacerbations.

This continuum model has an important implication for the present hypothesis. If inflammation is not episodic but rather is constant, then the exposure is the cumulative inflammatory burden, not the number of exacerbations. Patients with persistent *Haemophilus* dominance and a small community membership are more likely to have more cumulative exposure to the vasculature than patients with intermittent severe exacerbations and a larger community. Studies that have used exacerbation frequency as their exposure variable may thus be measuring the inappropriate exposure metric, which may account for the difficulty in demonstrating associations with vascular outcome.

This reasoning can be translated into an exposure variable that can be tested. We suggest that future longitudinal studies measure the total microbial load, instead of the number of exacerbations. This would involve serial sputum or bronchoalveolar samples, not just at exacerbation; quantification of pathogen abundance by quantitative culture or quantitative PCR in addition to relative abundance by sequencing; and repeated measurements over time (e.g., area under the burden-versus-time curve) to provide a single cumulative exposure metric for each individual. That measurement then might be correlated with longitudinal pulmonary vascular outcomes in the cohort design described in Section 8.4 and directly compared with the count of exacerbations as an exposure. We emphasise that cumulative microbial burden is at present a research proposal: it has not been operationalised, validated or related to vascular outcomes in COPD and no biomarker of this kind currently exists.

### 6.3. Host Responses That Allow Persistence

Several levels of airway microbiome–immune crosstalk are impaired in COPD [33,43]. The phagocytic function of macrophages is impaired. Mucosal antibody responses are compartmentally deficient, as demonstrated by the NTHi-specific IgG1 results [24]. Interferon responses to viral infection are reduced and this is one of the factors that underlie the increased susceptibility observed in experimental rhinovirus studies [14]. In addition, corticosteroid treatment, especially inhaled, alters host defence and is linked to the risk of pneumonia [13].

These defects are mostly a result of the already existing COPD and thus raise the issue of reverse causation: the immune defect that allows for colonisation is caused by the disease whose progression colonisation is suggested to accelerate. Longitudinal sampling from early disease is needed to separate the two and this is not available in the field.

### 6.4. The Gut–Lung Axis

The effects of microbes on the pulmonary vasculature are not limited to the airway. Gut communities are changed in COPD [34,35], gut dysbiosis is associated with disease development in experimental models [35,44] and gut composition is associated with lung function decline [45]. This relationship is now known for being bidirectional [46,47,48,49,50,51,52,53,54,55].

Mechanistic links are more clearly defined in PAH than in COPD-PH but are directly relevant. Loss of diversity and depletion of short-chain fatty acid (SCFA)-producing bacteria are associated with impaired gut barrier integrity, which permits translocation of bacterial lipopolysaccharide (LPS) into the systemic circulation [56]. Circulating LPS is recognised through the canonical CD14–MD-2–TLR4 complex, leading to downstream cytokine production and increased endothelial permeability [56,57]. HMGB1 is an endogenous danger-associated signal that can bind LPS and amplify TLR4-dependent responses; it is a modulator of this pathway rather than the mediator of LPS recognition. Trimethylamine N-oxide (TMAO) reaches the circulation by a different route and the two should not be conflated: gut bacteria metabolise dietary choline, phosphatidylcholine and L-carnitine to trimethylamine (TMA), which is absorbed and then oxidised to TMAO predominantly in the liver by host flavin-containing monooxygenases. Thus, bacteria produce the precursor but not TMAO itself. Pulmonary arterial hypertension patients have a proven pro-inflammatory gut microbiome [58] and a decrease in microbiota-derived SCFAs is associated with disease pathogenesis [59].

Conversely, experimental studies have demonstrated that microbiome-derived metabolites can directly influence pulmonary vascular remodelling. Butyrate, a microbial fermentation product that is a histone deacetylase inhibitor, reduces pulmonary vascular remodelling and inflammation in rodent hypoxia-induced pulmonary hypertension and has been shown to have an effect on vascular permeability and endothelial barrier function [60]. SCFAs exert their effects via G protein-coupled receptors such as GPR41, GPR43 and GPR109a and directly affect endothelial function [57,61].

This is a second microbial pathway to the pulmonary vasculature that is not dependent on the burden of pathogens in the airways—and one for which interventional evidence of causation exists in animals, although not in humans.

An important limitation should nevertheless be acknowledged. Gut dysbiosis is typically described as an “upstream” contributor to pulmonary disease. Much of the human evidence is equally compatible with the opposite: systemic inflammation leads to changes in gut barrier function, hypoxaemia leads to changes in intestinal perfusion and the drugs used in COPD, especially antibiotics and corticosteroids, have a profound effect on gut communities. There are several reasons why gut microbiota is altered in a severe COPD patient, which are not related to the gut–lung axis. Interventional animal studies therefore have more weight here than cross-sectional human comparisons because they establish the direction of effect.

### 6.5. Methodological Limitations of Microbiome Evidence

The microbiome evidence summarised above has methodological limitations which are relevant for causal interpretation. The lower airway is a low biomass environment and reagent and laboratory contamination can account for a significant percentage of the sequences recovered if negative controls are not processed and reported. Sampling route matters: spontaneously expectorated sputum is not free from oral and upper-airway carry-over, while BAL is not as susceptible to this issue but it is only sampling a small region of a heterogeneous lung and is infrequently repeated longitudinally. Amplicon and metagenomic sequencing only identifies nucleic acid, so relative-abundance data alone establish neither absolute microbial burden, nor organism viability, nor active pathogen behaviour; each of these requires separate measurement. Sequencing data are also compositional, meaning that an increase in the relative abundance of one taxon can be due to a decrease in another and that conclusions about bacterial burden must be made in parallel with quantitative measurement of absolute load.

Confounding is also a major concern. Antibiotic use (intermittent or long-term) directly affects airway and gut communities. Inhaled and systemic corticosteroids affect host defence and are linked to changes in airway bacteria and risk of pneumonia. Bronchiectasis is a common feature of advanced COPD and is often not diagnosed and it has its own microbiological profile and vascular and inflammatory associations. Gut community structure, output of metabolites such as SCFA and TMA production are influenced by diet and are rarely documented in respiratory cohorts. Both current and cumulative smoking exposure have an independent impact on airway ecology and endothelial function. The intensity of treatment depends on the severity of the disease and sampling feasibility and the likelihood of vascular disease occurring simultaneously make it possible that associations between microbial composition and pulmonary vascular measures in cross-sectional studies could be explained by these shared causes and none of the published human data preclude that explanation.

## 7. Molecular Pathways from Microbial Recognition to Vascular Remodelling

**Figure 4:**
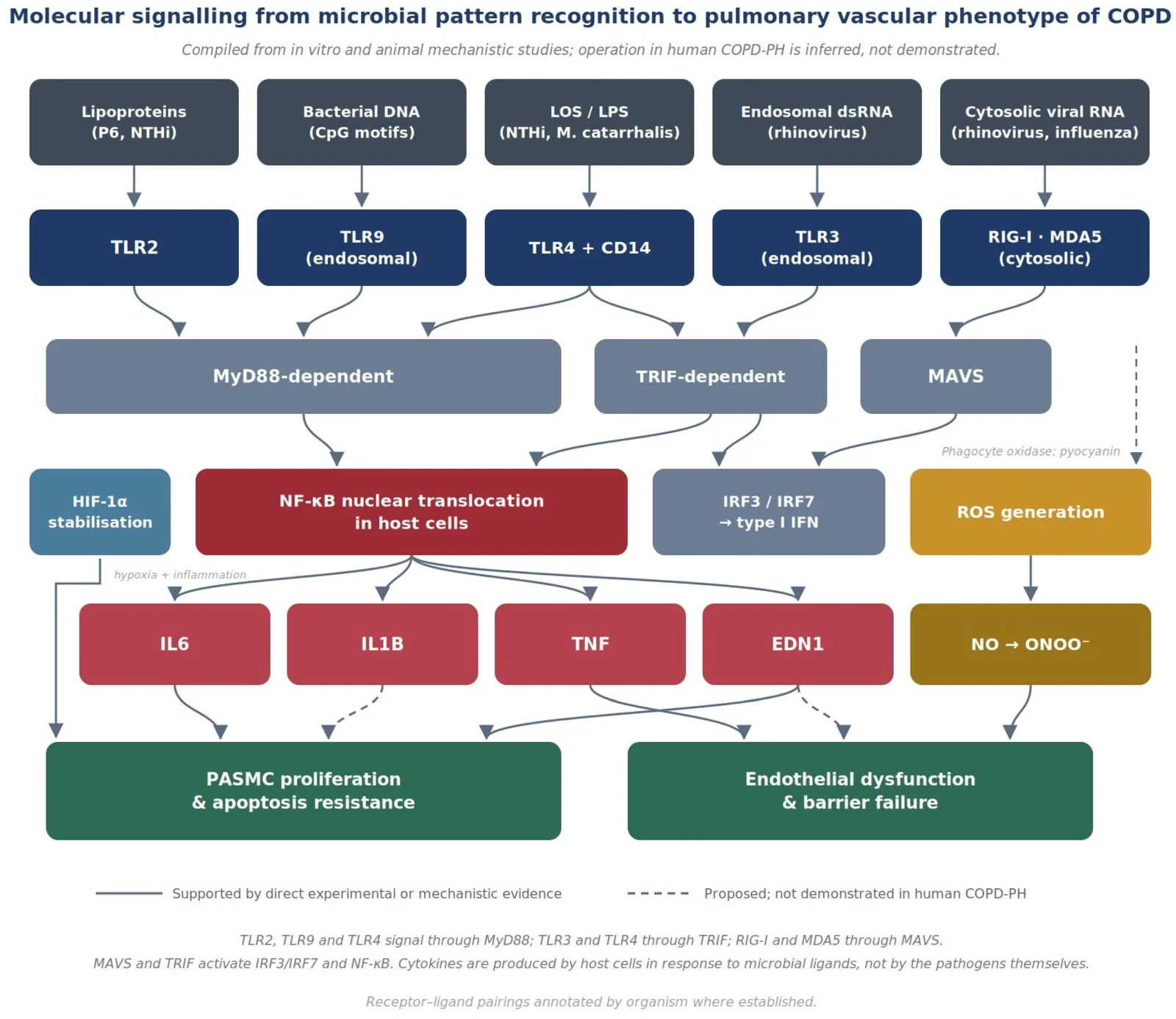
Molecular signalling from microbial pattern recognition to pulmonary vascular phenotype of COPD. Schematic of intracellular signalling after microbial recognition, based on published mechanistic studies and adapted to correct the adaptor assignments. Bacterial lipoproteins bind to TLR2, lipooligosaccharide and lipopolysaccharide are recognised by the CD14–MD-2–TLR4 complex, unmethylated CpG DNA binds to endosomal TLR9, double-stranded RNA binds to endosomal TLR3 and cytosolic viral RNA binds to RIG-I and MDA5. TLR2, TLR9 and TLR4 signal through MyD88; TLR3 and TLR4 signal through TRIF; RIG-I and MDA5 signal through the mitochondrial adaptor MAVS. MyD88- and TRIF-dependent signalling converge on NF-κB nuclear translocation, TRIF and MAVS also activate IRF3 and IRF7. Cytokine and mediator transcription (*IL6*, *TNF*, *IL1B*, *EDN1*) occurs in host epithelial, myeloid and endothelial cells in response to microbial ligands; the pathogens themselves do not produce these mediators. Parallel arms depict ROS generation with NO scavenging to peroxynitrite and HIF-1α stabilisation occurring under both hypoxic and inflammatory conditions. Pathways converge on pulmonary artery smooth muscle cell proliferation and endothelial dysfunction. Receptor–ligand pairings are annotated by organism where established. Relationships shown derive from in vitro and animal studies; their operation in human COPD-PH is inferred rather than demonstrated.

As described in previous sections, microbial exposure initiates inflammatory responses that may subsequently influence pulmonary vascular remodelling. This section outlines the potential pathways by which that inflammation could progress to and alter the pulmonary vasculature (Figure 4; Table 4).

### 7.1. Pattern Recognition and NF-κB

TLRs are expressed on epithelium, alveolar macrophages and endothelium and recognise microbial products: TLR2 for lipoproteins, TLR4 for LPS and LOS, TLR9 for bacterial DNA, TLR3 and TLR7/8 for viral nucleic acids [21,26]. These receptors do not have a common adaptor. TLR2, TLR9 and TLR4 signal through MyD88; TLR3 and, in a second wave, TLR4, signal through TRIF; and the cytosolic RNA sensors RIG-I and MDA5 signal through the mitochondrial adaptor MAVS. MyD88- and TRIF-dependent signalling converges on NF-κB, while TRIF and MAVS additionally activate IRF3 and IRF7 and the type I interferon response [21,26].

These signals converge on NF-κB, which leads to the transcription of *IL6*, *TNF*, *IL1B*, adhesion molecules and, importantly for vascular tone, *EDN1*. Pulmonary artery endothelial cells (PAECs) are TLR-expressing and directly respond to microbial products. The vasculature may directly participate in the inflammatory response that is the target of cytokines produced elsewhere.

### 7.2. The Cytokine Axis

IL-6 is among the mediators most consistently associated with pulmonary vascular disease. It stimulates pulmonary artery smooth muscle cell proliferation and resistance to apoptosis and IL-6 overexpression is sufficient to produce pulmonary hypertensive remodelling in animal models [12,16]. Although these are experimental observations, they offer mechanistic plausibility for a similar pathway in COPD-PH, which has not been directly causally proven in human COPD-PH. It is increased in COPD in the stable state and increases significantly during exacerbation [3].

TNF-α has been shown to inhibit the function of endothelial nitric oxide synthase, increase the expression of adhesion molecules and induce endothelial cell apoptosis [12,16].

Inflammasome activation is a mechanism of IL-1β production and it is known to be activated by pneumolysin and NTHi [24]. It is associated with the bacterial load in sputum [25] and is responsible for additional production of IL-6, thus further amplifying the axis.

### 7.3. Endothelin-1 and Nitric Oxide

Inflammation directly affects the vasoactive imbalance that is present in COPD-PH, which is an increase in ET-1 and a decrease in nitric oxide.

The transcription of *EDN1* is NF-κB-responsive, meaning that inflammatory stimulation leads to increased production of ET-1. ET-1 exerts its effects via ETA receptors on smooth muscle, causing vasoconstriction and mitogenesis [7,16]. Persistent ET-1 elevation acts both as a potent vasoconstrictor and as a mitogenic growth factor, producing both functional and structural vascular changes.

NO availability is decreased by multiple inflammation-related mechanisms, including decreased eNOS expression and activity, eNOS uncoupling due to depletion of tetrahydrobiopterin, scavenging by superoxide to form peroxynitrite and accumulation of asymmetric dimethylarginine, a competitive inhibitor. Chronic infection is an oxidative environment that promotes each.

### 7.4. Oxidative Stress and HIF Signalling

Reactive oxygen species are produced by activated neutrophils and macrophages, by mitochondrial dysfunction [62] and, in the case of *P. aeruginosa* colonisation, directly by pyocyanin. They are involved in endothelial damage, scavenging of NO and activation of redox-sensitive transcription factors [63].

**Figure 5:**
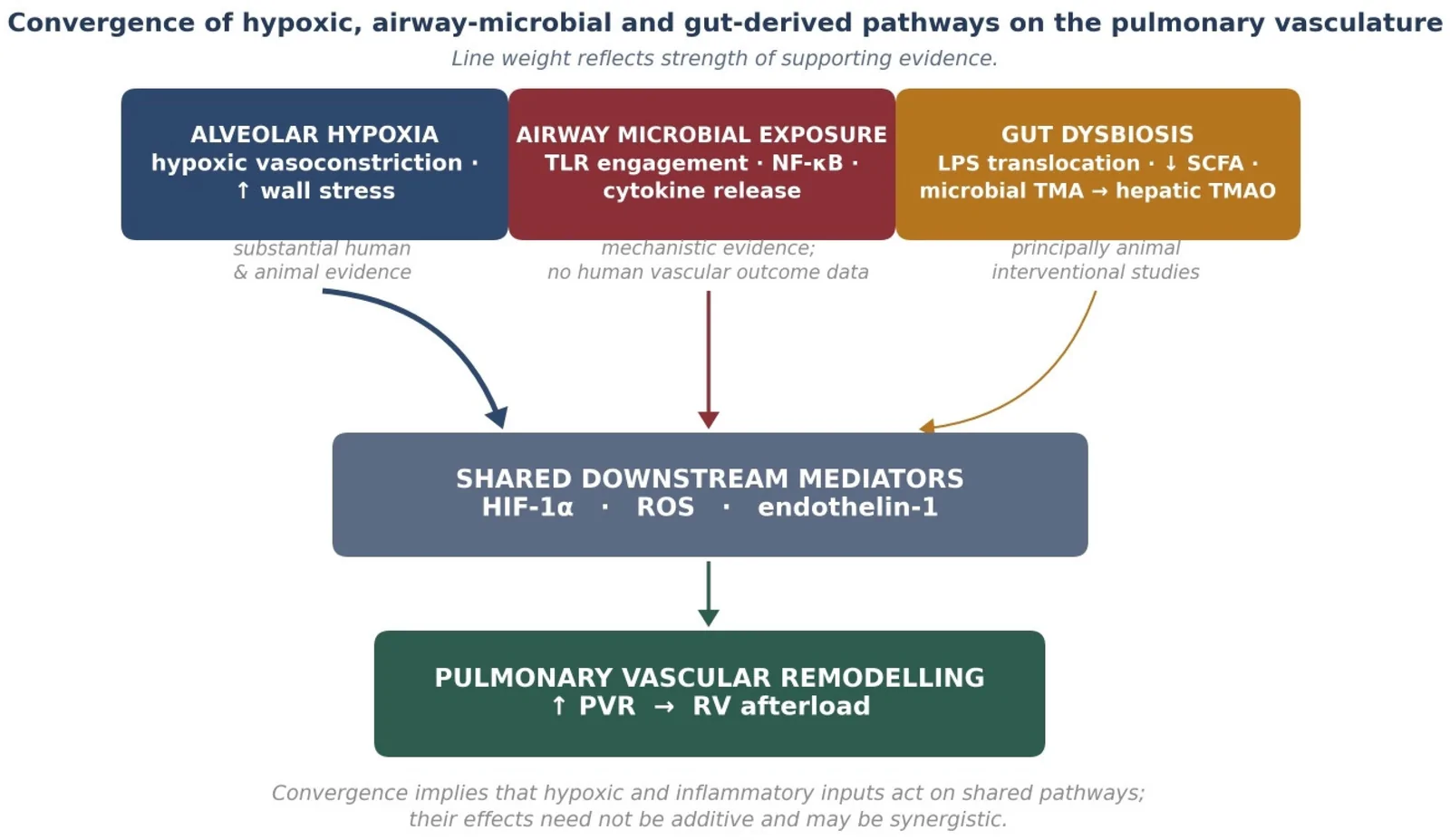
Convergence of hypoxic, airway-microbial and gut-derived pathways on the pulmonary vasculature. Schematic showing three proposed inputs converging on shared downstream mediators. Alveolar hypoxia acts via hypoxic pulmonary vasoconstriction, HIF-1α stabilisation and increased wall stress. Airway microbial exposure acts via TLR engagement, NF-κB activation and cytokine release. Gut dysbiosis acts via lipopolysaccharide translocation, depletion of short-chain fatty acids and the trimethylamine (TMA) pathway; gut bacteria generate TMA from dietary precursors, TMA is absorbed from the intestine and is then oxidised to trimethylamine N-oxide (TMAO) by host hepatic flavin-containing monooxygenases, so TMAO itself is not translocated from the gut. Convergence occurs at HIF-1α, ROS and ET-1. The hypoxic pathway is supported by substantial human and animal evidence; the airway-microbial pathway is supported by mechanistic but not human vascular outcome data; the gut-derived pathway is supported principally by animal interventional studies. Line weight reflects strength of supporting evidence.

The most obvious overlap between infection-driven and hypoxia-driven pathways is hypoxia-inducible factor (HIF) signalling. HIF-1α stabilisation is not only induced by hypoxia but also by inflammatory stimulation in the absence of oxygen. Hypoxic pulmonary vasoconstriction leads to innate immune activation through HIF-1α and the increased wall stress it causes leads to further remodelling [6]. Thus, inflammation and hypoxia do not work in parallel but rather on a common downstream pathway and their effects do not necessarily have to be additive and can be synergistic (Figure 5).

### 7.5. Growth Factors and Structural Remodelling

TGF-β stimulates extracellular matrix deposition, smooth muscle proliferation and endothelial-to-mesenchymal transition [16]. VEGF is in a more ambiguous position: it is necessary for endothelial maintenance and its reduction is thought to lead to capillary rarefaction in emphysema but its dysregulated signalling is thought to lead to plexiform lesions in severe pulmonary hypertension [7,46]. Further endothelial dysfunction may occur due to alveolar hypoxia via VEGF-dependent mechanisms [7].

### 7.6. Barrier Failure, Thrombosis and Microvascular Loss

Endothelial barrier integrity is compromised by pneumolysin pore formation, TLR4-mediated permeability increases and TMAO acting through HMGB1/TLR4 [57]. Perivascular oedema and infiltration of inflammatory cells into the vessel wall occur due to increased permeability.

Infection also induces a prothrombotic state by expression of tissue factor, activation of platelets and inhibition of fibrinolysis. In situ thrombosis is a factor in vascular obliteration and is reported in severe pulmonary hypertension [16]; in COPD, destruction of the capillary surface by emphysema adds to this loss of vascular surface [7].

### 7.7. How Does Airway Inflammation Get to the Vasculature?

An important unresolved question is how airway inflammation reaches the pulmonary vasculature. The inflammatory signal needs to reach the vessel wall for airway infection to impact pulmonary circulation.

There are three possible routes. The simplest is anatomical proximity: small pulmonary arteries are adjacent to terminal bronchioles and peribronchiolar inflammation is close to the adventitia of the accompanying vessels and mediators have to travel only a short distance. The second is systemic spillover—cytokines generated in the lung are detectable in the serum during exacerbation (increased serum IL-6 and C-reactive protein) and affect the endothelium throughout the pulmonary bed. The third is cellular trafficking, whereby activated monocytes and lymphocytes are recruited to the airway and then migrate to perivascular sites.

The relative contribution is not known but the distinction is testable. Diffuse change is predicted by systemic spillover and patchy change is predicted by proximity. Histological studies of the spatial distribution of vascular remodelling and airway inflammation may be able to distinguish between them. As far as we know, no one has done this.

## 8. Marker or Driver? A Critical Appraisal of Causal Inference

The key question is whether the microbial factors are playing a role in pulmonary vascular remodelling or are merely a consequence of the advanced disease and all that follows from the hypothesis hinges on the answer. The mechanistic material presented in Section 5, Section 6 and Section 7 provides evidence that plausible pathways from microbial recognition to vascular injury are present but does not provide evidence that these pathways are active in human COPD-PH. It should, therefore, be evaluated in the light of that evidence and the uncertainty it raises should be carried back into the reading of the preceding sections and not be assumed to have been overcome in those sections.

### 8.1. The Case for a Causal Contribution

Temporality. Airway dysbiosis can be identified in frequent exacerbators during periods of clinical stability, not just during acute events [9]; because that comparison was cross-sectional and used prior-year exacerbation history, it demonstrates association rather than temporality. Dysbiotic signatures have been demonstrated to precede accelerated lung function decline [40] and thus microbial change is not just a consequence of the acute episode. Vascular abnormalities also occur early, prior to hypoxaemia [5,7], and there is room for another initiating factor.

Biological plausibility. Pathways are co-shared. TLR2 and TLR4 recognise bacterial lipoproteins and lipooligosaccharide (LOS), respectively, and activate NF-κB and induce the production of IL-6, TNF-α and IL-1β [21,26]. These cytokines have known effects on endothelial function and IL-6 has been implicated in pulmonary vascular remodelling across aetiologies of PH, principally in experimental models and in human pulmonary arterial hypertension [12,16]. Each individual step between pathogen recognition and vascular pathology has been demonstrated in some experimental system; the sequence as a whole has not been demonstrated in human COPD-PH.

Dose–response. Frequent exacerbators have a more rapid decline in lung function than infrequent exacerbators [2,3]. A similar gradient for vascular outcomes would add a lot to the causal argument. There is no evidence at this time on this particular issue.

Experimental manipulation. Modification of the gut microbiota by antibiotics or diet alters the pulmonary vascular phenotype in rodent models of pulmonary hypertension [64,65] and butyrate supplementation has been shown to reduce hypoxia-induced vascular remodelling and inflammation [60]. Modification of microbiota inhibits the development of PAH in rodents [64]. These experiments demonstrate that the vascular outcome can be modified by modifying the microbiota in animals, which cannot be determined by any observational human study. Whether the same holds in human COPD-PH remains to be demonstrated.

Compositional specificity. Frequent exacerbators have sputum microbiome signatures that are distinguishable during stability, with decreased alpha diversity and different beta diversity patterns [9]. Severity and outcome are worse with *Streptococcus*-dominant and *Haemophilus*-dominant profiles [9,25], although both observations are cross-sectional and neither included vascular endpoints. Such specificity may not seem to be expected in a purely epiphenomenal relationship.

### 8.2. Limitations of the Current Evidence

Reverse causation. In advanced COPD, mucociliary clearance is impaired, there are changes in airway architecture and local immunity [21,43]. So patients with more severe disease are more susceptible to colonisation for reasons entirely downstream of their disease and the same patients have more vascular disease. No causal arrow from infection to vasculature is required—correlation follows.

Confounding by severity. This is the most significant one raised. Emphysema leads to the loss of capillary bed and loss of defence at the same time; hypoxaemia causes vasoconstriction and also affects the function of neutrophils; smoking damages the endothelium and affects mucosal immunity. There is an association that is caused by multiple common causes.

Collinearity with decrease in lung function. Exacerbations are associated with FEV_1_ decline and FEV_1_ decline is associated with vascular disease. Studies measuring only one of these are not able to determine whether infection has an independent effect on the vasculature or not.

Absence of adjusted human data. There are no published cohorts that have studied exacerbation frequency in relation to pulmonary vascular endpoints, adjusted for age, smoking exposure, FEV_1_, hypoxaemia and comorbidity. Human evidence is associational until such analyses are available.

Restrictions of animal models. The mechanism of rodent hypoxic pulmonary hypertension is different from that of human COPD-PH; there are also differences regarding the time course and lack of parenchymal destruction [6,60]. The fact that butyrate attenuates remodelling in a hypoxic rat does not prove that microbiome modulation would affect vascular outcome in a patient with emphysema.

### 8.3. Assessment

The available evidence provides biological plausibility. However, biological plausibility should not be interpreted as proof of causation (Table 5).

The causal argument does not stand on its own on the grounds of any one observation. It is based on three things: exacerbation frequency is variable within severity strata; microbial signatures during stability are associated with a history of frequent exacerbations; and experimental manipulation of the microbiota alters vascular phenotype in animals. One objection to this has never been met and that is that severity confuses everything and no human study has been able to make the proper adjustment for it.

Therapeutic implications depend directly on whether microbial exposure contributes causally to vascular remodelling. If infection is a sign of late disease, prevention will have a positive impact on symptoms and hospitalisation without affecting the vascular trajectory. These possibilities cannot be distinguished at present.

### 8.4. What Would Test the Hypothesis

Rather than making general recommendations for future research, we propose specific study designs capable of directly testing the remaining uncertainties and hypotheses.

What is needed is a prospective cohort of patients with COPD without pulmonary hypertension at baseline (defined by right heart catheterisation or serial echocardiography) followed for at least five years with exacerbations documented prospectively and microbiologically confirmed and with multivariable adjustment for age, sex, smoking pack-years, FEV_1_, arterial oxygen tension, body mass index and cardiovascular comorbidity. The result would be incident pulmonary hypertension or change in pulmonary vascular resistance. Such adjustment would not affect a dose–response relationship, which would greatly support the causal argument.

Two complementary approaches should be considered. Directly addressing confounding by using genetic variants associated with exacerbation susceptibility in Mendelian randomisation. More immediately, experimental human data could be obtained from post hoc analysis of vascular outcomes in existing RCTs of exacerbation prevention, where a reduction in the incidence of pulmonary hypertension under macrolide prophylaxis or vaccination would substantially strengthen the causal argument, although such post hoc analyses would remain hypothesis-generating. The trials required for such an analysis have already been performed [49,50,66,67] and their pulmonary vascular outcomes have never been reported.

## 9. Right Ventricular Consequences

Pulmonary vascular remodelling increases right ventricular afterload and the right ventricle initially adapts through myocardial hypertrophy, increasing wall thickness to maintain cardiac output before progressive dilatation and right ventricular failure develop.

The right ventricle is not well suited for this function. It is thin-walled and crescentic and is adapted to a low-resistance circulation and is not very sensitive to volume loading but is very sensitive to pressure loading. Normally, its coronary perfusion takes place during the entire cardiac cycle but during systole, when the intracavitary pressure increases, the coronary perfusion is reduced and the hypertrophied ventricle is subjected to increased demand and decreased supply. Ventricular interdependence is an additional burden, as right ventricular dilatation causes the interventricular septum to shift leftward, causing a decrease in left ventricular filling, which is unrelated to left ventricular contractility, and hyperinflation in COPD decreases preload.

The acute afterload stress imposed by exacerbations is due to the physiological reasons outlined in Section 4 and is imposed on this chronically loaded ventricle. Each episode is thus a haemodynamic event as well as a respiratory event and repeated episodes may accumulate—although there is no direct evidence of this longitudinally.

As with practice, assessment limits research. Pressure estimates are less reproducible in hyperinflated patients than right ventricular functional indices (tricuspid annular plane systolic excursion, fractional area change, tissue Doppler velocities and strain measures) and may be better endpoints for the longitudinal work identified in Section 8.4. They also directly measure the consequence of interest, not from an estimated pressure.

This is directly related to the hypothesis formulated in Section 8. The right ventricle is the downstream manifestation of pulmonary vascular remodelling and right ventricular measures are appropriate endpoints for the prospective study proposed in Section 8.4, if the cumulative exposures to microbes and inflammation are the cause of pulmonary vascular remodelling. If there is a link between cumulative microbial burden and vascular progression, it seems plausible to consider TAPSE, RV FAC, RV strain and RV dimensions as serial non-invasive endpoints, linked, to a possible extent, to invasive pulmonary haemodynamics and to clinical deterioration. These measurements are more reproducible than echocardiographic pressure estimation in hyperinflated patients and they are a direct index of the consequence of interest. It is important to note that there is no evidence that any microbial or inflammatory exposure causes right ventricular dysfunction in COPD and the suggestion here is that right ventricular assessment provides a feasible approach to testing this.

## 10. Biomarkers

There is no validated biomarker for infection-associated pulmonary vascular injury in COPD. We state this simply because the candidates available are from related clinical contexts and none have been evaluated for this use.

NT-proBNP is a measure of ventricular wall stress and is of prognostic importance but is not specific to pulmonary vascular disease and does not provide information on aetiology. C-reactive protein and IL-6 are markers of systemic inflammation and are useful in predicting outcome but do not differentiate infectious from other causes of inflammation or suggest vascular involvement. ET-1 is mechanistically linked to the pathology of interest but standardisation of assays is restricted and it has not been assessed as a marker of vascular change in the context of infection. Asymmetric dimethylarginine is a marker of the NO pathway and is increased in COPD but there is limited data on COPD-PH.

Endothelial injury markers (circulating endothelial cells, endothelial microparticles, von Willebrand factor, soluble adhesion molecules) are more direct markers of the process of interest. Microparticles are elevated in smokers with normal lung function [5] and are likely to be sensitive enough to detect early injury but their behaviour during and after exacerbation has not been characterised.

The most conceptually attractive markers are those derived from the microbiome, which index the exposure itself, not its downstream consequences. Functional signatures of the airway microbiome in COPD have been identified using multi-omic meta-analysis [47] and bronchoalveolar lavage microbiome–metabolome analysis has identified candidate diagnostic markers [48]. The lung microbiome has been proposed as a biomarker and therapeutic target and the rationale for this has been extensively discussed [42]. None of these have been tested for pulmonary vascular endpoints.

A useful panel would be a combination of an exposure marker, an endothelial injury marker and a haemodynamic marker, validated against catheterisation confirmed outcomes in a cohort as described in Section 8.4. One methodological caution applies to any such panel: where a marker forms part of the definition of the outcome against which it is tested, incorporation bias inflates its apparent performance. For any pulmonary equivalent, an endpoint would have to be established that is independent of the markers being assessed. There would be two considerations that would govern such an effort. Timing is important: markers measured during exacerbation are markers of the acute event, markers measured during stability are markers of the chronic state and which is more important in predicting vascular outcome is not clear—serial measurements in both states would be required. Specificity is also important: any candidate should be able to differentiate pulmonary from systemic vascular injury, as patients with COPD have a high prevalence of systemic atherosclerosis and most endothelial markers are not specific for vascular beds.

## 11. Prevention: Vaccination, Antimicrobial Strategies and Microbiome Modulation

If there is a causal link between microbial factors and vascular remodelling, then interventions that decrease microbial burden should slow vascular progression. That inference has not been tested but the interventions themselves show evidence for their primary indications (Table 6).

### 11.1. Vaccination

A Cochrane review of randomised trials found that inactivated influenza vaccination reduced the total number of exacerbations in COPD, an effect attributable to influenza-related exacerbations occurring more than three weeks after vaccination. The few and small trials that contributed to that estimate were randomised but the analyses of hospitalisation and of mortality were underpowered and did not show an effect, and a reduction in COPD mortality should not therefore be described as high-quality randomised evidence [49]. Pneumococcal vaccination has been linked to a decrease in pneumonia and hospitalisation in COPD, most pronounced in younger patients and in patients with more severe airflow limitation and the extent of the benefit is dependent on vaccine type and serotype coverage; a mortality benefit has not been demonstrated [50]. Recently, RSV vaccines have been developed for older adults and are a potential candidate for inclusion, although there is limited data on outcomes in people with COPD. COVID-19 vaccination is also recommended for people with COPD in line with current GOLD and national public-health guidance [1].

None of these trials focused on vascular outcomes. It is not known whether vaccination affects pulmonary haemodynamics but trials have already been conducted that have the required exposure data.

### 11.2. Macrolide Prophylaxis

Among available preventive interventions, long-term macrolide therapy has the strongest evidence base.

In the MACRO trial, 1142 patients at a high risk of exacerbation were randomised to azithromycin 250 mg daily or placebo for 1 year, which increased the median time to first exacerbation from 174 to 266 days and decreased exacerbation rate [66]. Nine RCTs with 1666 patients were included in a meta-analysis, which showed that macrolides decreased exacerbation rates (RR 0.70, 95% CI 0.56–0.87) and that 6–12 months of erythromycin or azithromycin was effective by both daily and intermittent dosing but there was no significant difference in hospitalisation or all-cause mortality [67].

The mechanism seems to be much more immunomodulatory than antimicrobial, which is directly relevant here [68]. It implies that it is not necessary to kill organisms to be beneficial but to reduce the host response to the presence of microbes. If so, the target of therapy is the host response rather than the organism. It is important to note that there is no evidence of pulmonary vascular benefit of macrolide therapy in any population; the vascular rationale outlined here is speculative and the proven indication is exacerbation prevention in selected frequent exacerbators.

These benefits must be balanced against well-recognised risks. MACRO reported decrements in 25% versus 20% and almost doubled macrolide-resistant colonisation [66]; meta-analyses showed a trend towards more adverse events (OR 1.55, 95% CI 1.003–2.39) [67]; QT prolongation is a cardiovascular risk. However, resistance results are not consistent: COLUMBUS, which employed targeted metagenomics for *ermB*, *ermF* and *mefA*, did not detect any increase in the acquisition of resistance genes over 12 months [69]. The BACE trial expanded the evaluation to the acute hospitalised setting [70]. Prophylaxis is therefore only recommended for selected frequent exacerbators and the benefit-to-resistance ratio is still a matter of debate.

### 11.3. Microbiome-Directed Approaches

Microbiome-targeted interventions are still in the early stages. A pilot randomised controlled trial has evaluated influenza–pneumococcal vaccination, oral probiotics and inhaled amikacin in combination, with explicit microbiome-modulating intent [71]. The use of probiotics, prebiotics and dietary interventions have been suggested for COPD, mainly on the basis of the gut–lung axis [72,73] and antibiotic-based microbiota-targeted therapy has been investigated for advanced pulmonary hypertension [65]. The metabolites of commensal organisms with antioxidant properties have also been studied as modulators of disease progression [74].

The biological rationale is compelling and preclinical studies provide encouraging support [60,64]. However, robust clinical evidence demonstrating vascular benefit in humans remains unavailable.

### 11.4. Non-Pharmacological Measures

Quitting smoking is still the best treatment for slowing the progression of COPD. Pulmonary rehabilitation increases exercise capacity and decreases hospitalisation [75]. Long-term oxygen therapy in patients with severe resting hypoxaemia is the only intervention that has been shown to have a beneficial effect on pulmonary haemodynamics and thus on the progression of pulmonary artery pressure and is the benchmark against which other vascular-directed interventions should be judged.

## 12. Vascular-Directed Therapy: Rationale Versus Evidence

Physiological rationale and clinical evidence are conflicting here and care must be taken (Table 7).

### 12.1. The Rationale

ET-1 is increased and nitric oxide availability is decreased in patients with COPD-PH [7,17]. Phosphodiesterase-5 (PDE-5) inhibitors enhance NO–cGMP signalling and endothelin receptor antagonists inhibit ETA-mediated vasoconstriction and mitogenesis. Both aim to show obvious abnormalities and the rationale is why these agents were used.

### 12.2. The Evidence

The clinical evidence is insufficient to support that rationale in practice, and the guideline position is more nuanced than an outright ban. The 2022 ESC/ERS guidelines do not make a Class III recommendation against phosphodiesterase-5 inhibitors for the entire Group 3. For pulmonary hypertension associated with interstitial lung disease, phosphodiesterase-5 inhibitors may be considered in severe PH with individualised decision-making at a pulmonary hypertension centre and are not recommended when PH is non-severe; ambrisentan is not recommended in idiopathic pulmonary fibrosis and riociguat is not recommended in PH associated with idiopathic interstitial pneumonias. For COPD-PH specifically, no pulmonary arterial hypertension therapy is recommended: the guidelines advise optimisation of the underlying lung disease and hypoxaemia and referral of patients with suspected severe PH to a pulmonary hypertension centre for individualised assessment, preferably within clinical trials [4]. These distinctions matter for the present argument, because evidence generated in PH associated with interstitial lung disease cannot be transferred to COPD-PH. Phosphodiesterase-5 inhibitors are nevertheless prescribed in this population in routine practice, well beyond what the evidence supports [76].

Harm is caused by a known and predictable mechanism. Homeostatic hypoxic pulmonary vasoconstriction (HPV) is a mechanism that redistributes blood flow away from poorly ventilated lung units. Systemic vasodilators block this response and lead to increased perfusion of poorly ventilated areas and further exacerbation of the ventilation–perfusion mismatch; this has been shown experimentally in isolated pulmonary arteries and in rat models [77] and reviewed for the drug class as a whole [78]. In COPD-PH itself, a randomised dose-comparison study of acute oral sildenafil in 20 patients reduced mean pulmonary arterial pressure at rest and during exercise but lowered arterial oxygen tension at rest by approximately 6 mmHg, because perfusion of units with a low V′/Q′ ratio increased [79].

Differences in outcomes vary instructively by underlying disease. Sildenafil has been reported to improve V′/Q′ matching and to increase arterial oxygen tension in pulmonary fibrosis [78], and ex vivo work on pulmonary arteries from patients with pulmonary fibrosis and pulmonary hypertension is consistent with a preserved vasodilator response in that setting [80]; the effect on oxygenation is the opposite in COPD-PH [79]. So extrapolation between Group 3 aetiologies is unsafe—not just for this class of drug.

The PERFECT trial of inhaled treprostinil in COPD-PH was prematurely stopped due to an increase in serious adverse events and there was suggestive evidence of an increase in mortality but no evidence of benefit on the primary endpoint [81]. The use of pulmonary vasodilators in patients with COPD-PH confirmed by right heart catheterisation has been described in a small real-world case series separately [82]. Such series are not comparable to randomised evidence and any apparent benefit in such series is easily explained by confounding by indication. Although there is a similar rationale, endothelin receptor antagonists have failed to demonstrate benefit in COPD. In a randomised, double-blind, placebo-controlled trial in 30 patients with severe or very severe COPD without severe pulmonary hypertension at rest, 12 weeks of bosentan did not improve six-minute walking distance and was accompanied by a fall in arterial oxygen tension, a widened alveolar–arterial oxygen gradient and worse health-related quality of life [83]. The randomised, placebo-controlled RISE-IIP study of riociguat in pulmonary hypertension (PH) due to idiopathic interstitial pneumonias (IIP) was terminated early because of a higher incidence of deaths, serious adverse events (SAEs) and adverse-event-related discontinuations in the riociguat arm, with no evidence of benefit [84].

### 12.3. How a Sound Rationale Can Cause Harm

It is good to be explicit about this, as the argument can be extended beyond the drugs in question.

Hypoxic pulmonary vasoconstriction is not a pathological process that needs to be corrected. It is a regulatory mechanism which is matched to perfusion and in a lung with heterogeneous ventilation it does real work. In pulmonary arterial hypertension, where ventilation is relatively uniform and the vasculopathy is diffuse, the inhibition of vasoconstriction is beneficial without cost. In COPD, where ventilation is highly heterogeneous, the same inhibition will cause a redistribution of blood flow to poorly ventilated units and thus affect gas exchange.

This is clearly illustrated by the fibrosis comparison. One drug had opposite effects on one physiological variable in two Group 3 conditions, most likely because the regional ventilation heterogeneity is less in fibrotic lungs than in emphysematous lungs. The mistake is to assume that if a mechanism is true, then it must have a clinical effect, without considering the disease context in which the mechanism is working.

This does not mean that vascular-directed therapy is useless. It does imply that the focus should be on the remodelling process and not on vascular tone. An agent that could reduce smooth muscle proliferation or matrix deposition without inhibiting hypoxic pulmonary vasoconstriction (HPV) would not be as liable; none is currently available for this indication. Highly preliminary haemodynamic and functional observations have been reported in an uncontrolled retrospective series of five patients receiving ensifentrine [85] but these data are insufficient to establish efficacy.

### 12.4. Position

Pulmonary vasodilators are not recommended for PH associated with COPD, except in specialist centres and preferably in clinical trials. The use in patients with the severe pulmonary vascular phenotype of COPD may represent a subset of patients in whom the balance is different and real-world series have reported their use in such patients [82] but this is not yet a standard practice.

**Figure 6:**
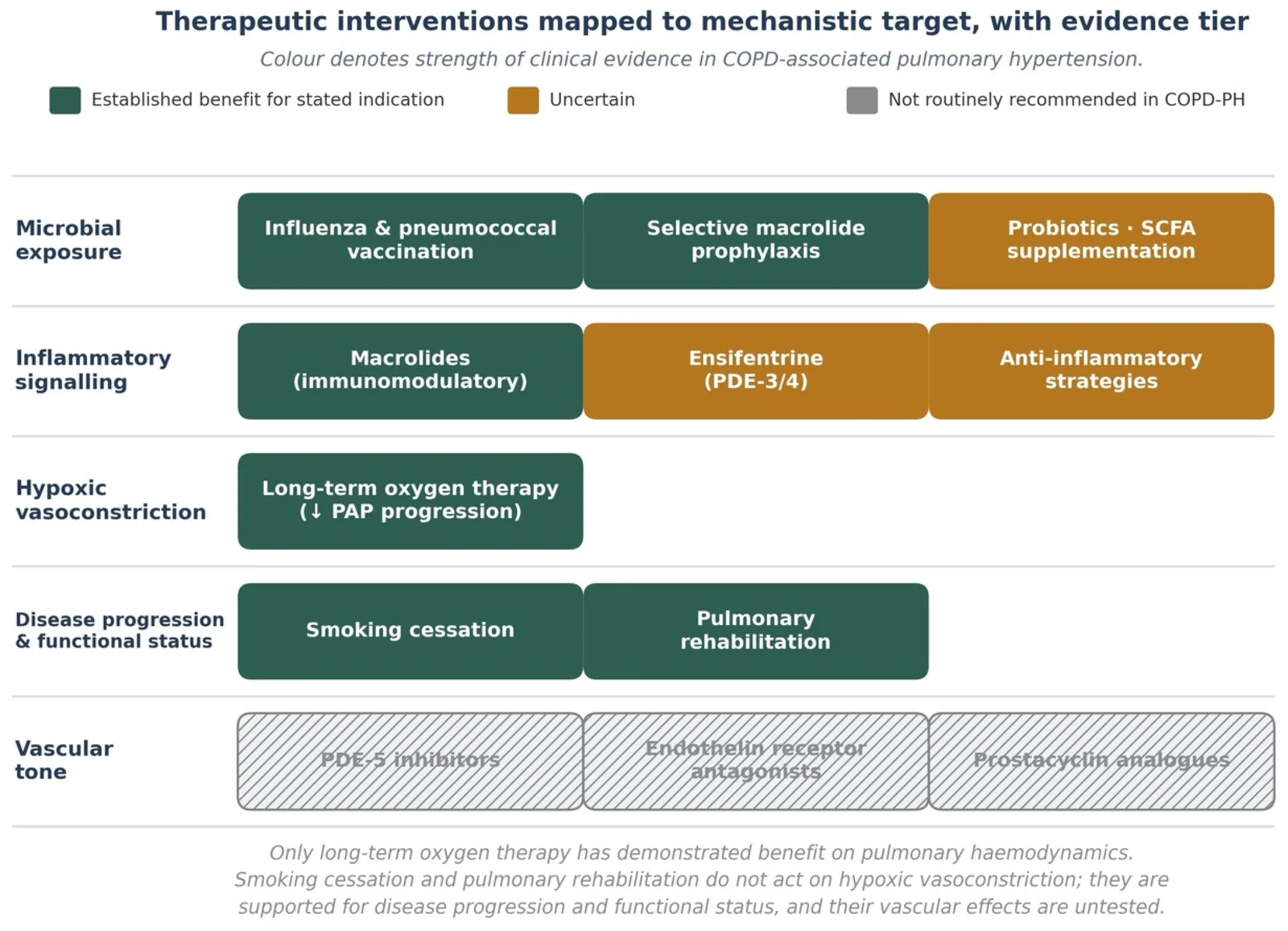
Therapeutic interventions mapped to mechanistic target, with evidence tier. Schematic positioning each intervention against the pathway node it targets, colour-coded by strength of clinical evidence in COPD-associated pulmonary hypertension. Established benefit for their stated indication (green): long-term oxygen therapy, influenza and pneumococcal vaccination, selective macrolide prophylaxis, pulmonary rehabilitation, smoking cessation. Uncertain (amber): microbiome-directed approaches including probiotics and short-chain fatty acid supplementation; ensifentrine. Not routinely recommended in COPD-associated pulmonary hypertension (grey, hatched): phosphodiesterase-5 inhibitors, endothelin receptor antagonists and prostacyclin-pathway agents. This tier is specific to COPD-PH and should not be interpreted as a blanket restriction for Group 3 as there are different recommendations for different underlying lung diseases and depending on the severity of the pulmonary hypertension: in PH associated with interstitial lung disease, phosphodiesterase-5 inhibitors may be considered in severe disease with individualised decision making at a pulmonary hypertension centre, while in COPD-PH no pulmonary arterial hypertension therapy is recommended and inhaled treprostinil increased serious adverse events in the PERFECT trial [81]. Where such therapy is contemplated in COPD-PH, decisions should be individualised in an expert pulmonary hypertension centre, preferably within clinical trials (Section 12). Smoking cessation and pulmonary rehabilitation are mapped to disease progression and functional status rather than to hypoxic vasoconstriction, which neither intervention modifies. Note that only long-term oxygen therapy has demonstrated benefit on pulmonary haemodynamics specifically; the remaining green-tier interventions are supported for exacerbation prevention or for disease progression and functional status, with vascular effects untested.

If the reasoning presented here holds true, the best therapeutic trajectory is upstream—where the microbial and inflammatory conditions that drive remodelling can be reduced—not downstream, simply by pharmacologically opening already remodelled vessels. The relationship between therapeutic targets, proposed mechanisms and the current level of supporting evidence is summarised in Figure 6.

## 13. Knowledge Gaps and Research Priorities

Mechanistic questions. Does infection-induced inflammation lead to vascular remodelling in the absence of hypoxia or only in the presence of hypoxia? Are some pathogens more vascular than others? Is it an accumulation of inflammatory burden or is it a number of exacerbations? Are there distinct pathways or common pathways for airway and gut dysbiosis?

Limitations of current human studies. Most of the evidence is cross-sectional, single-centre and has not been adjusted for the confounders listed in Section 8.2. Pulmonary vascular endpoints are infrequently measured and when measured, echocardiographic estimates are more common than catheterisation. Microbiome studies describe composition but do not include vascular measurements and exacerbation studies report frequency but not microbiology. The two studies have barely touched and that is the crux of the field and the obvious opportunity.

Longitudinal cohorts. The design specified in Section 8.4 has priority. The cumulative-burden hypothesis could also be directly compared to the exacerbation-count hypothesis by nested microbiome sampling during stability.

Multi-omics. Combining airway and gut metagenomics with metabolomics and host transcriptomics might reveal functional signatures instead of compositional associations [47,48]. This work has started in COPD in general and extension to vascular endpoints is a logical next step for future research.

Clinical trials. Three priorities deserve particular attention. The least expensive test of the causal hypothesis is retrospective analysis of pulmonary vascular outcomes in completed exacerbation-prevention trials, which do not require new recruitment [49,50,66,67]. This would be followed by a prospective trial of intensive exacerbation prevention with pulmonary haemodynamics as a pre-specified endpoint. In evaluation of microbiome-directed interventions, such as supplementation with butyrate or SCFA, the most experimentally supported candidate [60] would directly test the gut–lung route in patients with early vascular involvement.

Methodological priorities. Standardisation of methods of investigating airway microbiome in low-biomass samples with appropriate controls for contamination, agreed definitions of colonisation and infection and non-invasive vascular endpoints validated using parallel catheterisation in large cohorts.

## 14. Conclusions

Alveolar hypoxia is only a partial explanation for pulmonary hypertension in COPD. Microbial factors (chronic airway colonisation, dysbiosis of airway and gut communities and recurrent infectious exacerbation) are a potential additional contributor and the pathways linking microbial recognition to vascular remodelling are well described.

Correlation does not necessarily imply causation. The relationship between infection and vascular disease is complicated by severity and reverse causation is a plausible explanation for much of the observed association. Experimental data in animal models is more robust, especially in the gut–lung axis, but the applicability to human COPD-PH is unclear.

This uncertainty has ramifications for therapeutics. Vaccination, selective macrolide prophylaxis and pulmonary rehabilitation are each justified by their own proven benefits, which differ in evidence strength and are independent of any vascular effect; whether they slow vascular progression is unknown and testable. Although there is a coherent physiological rationale, the available clinical evidence is insufficient to support the routine use of pulmonary vasodilators in this population, and there is a specific mechanism of harm through ventilation–perfusion mismatch.

The hypothesis can be tested using existing clinical, microbiological and vascular assessment methodologies. Progress has been limited primarily because microbiological and pulmonary vascular research have evolved largely as separate fields. Including microbiological and pulmonary vascular endpoints in the same studies would permit direct testing of the hypothesis.

## Figures and Tables

**Table 1:** Haemodynamic definitions and classification relevant to COPD-associated pulmonary hypertension.

Parameter	Definition	Notes
Pulmonary hypertension	mPAP > 20 mmHg at rest	Threshold lowered from 25 mmHg in 2022 ESC/ERS guidelines
Pre-capillary PH	mPAP > 20 mmHg; PAWP ≤ 15 mmHg; PVR > 2 WU	Haemodynamic profile typical of COPD-PH
Group 3 PH	PH associated with lung disease and/or hypoxia	COPD-PH classified here
Non-severe COPD-PH	PVR > 2 to ≤5 WU	Common; must also satisfy pre-capillary criteria; largely tracks parenchymal disease
Severe COPD-PH	PVR > 5 WU	Uncommon; formal 2022 ESC/ERS category; disproportionate to airflow limitation
Pulmonary vascular phenotype of COPD	Vascular involvement out of proportion to obstruction	Concept, not a formal classification category

mPAP, mean pulmonary arterial pressure; PAWP, pulmonary artery wedge pressure; PVR, pulmonary vascular resistance; WU, Wood units. All thresholds follow the 2022 ESC/ERS guidelines. Non-severe COPD-PH requires PVR > 2 WU with PAWP ≤ 15 mmHg, so that patients who do not meet pre-capillary criteria are excluded from the category.

**Table 2:** Respiratory pathogens in COPD: recognition, virulence and potential vascular relevance.

Organism	Typical Role	Principal Recognition	Dominant Host Mediators Elicited	Persistence Strategy	Potential Vascular Relevance	Evidence Strength
Non-typeable *H. influenzae*	Most frequent bacterial isolate; ~50% of bacterial exacerbations; chronic colonisation	TLR2 (P6 lipoprotein), TLR4 (LOS), TLR6	TNF-α, IL-1β, IL-6	Phase variation, biofilm, intracellular residence	Sustained cytokine exposure; associated with frequent-exacerbator phenotype	Moderate (mechanism well characterised; vascular link inferred)
*S. pneumoniae*	Second most common; predominantly acute episodes	TLR2, TLR4, NLRP3 inflammasome	IL-1β, IL-6, complement activation	Capsule (anti-phagocytic)	Pneumolysin forms pores in host cell membranes and disrupts endothelial barrier integrity in vitro	Moderate
*M. catarrhalis*	Substantial minority of exacerbations	CD14–TLR4 (LOS); TLR2, TLR4, TLR9 (whole organism); partly NOD1	IFN-α2, CCL5, CCL3, CXCL10, CXCL11	Epithelial internalisation (trigger-like mechanism)	Alters epithelial tight junction components (*CLDN4*)	Moderate
*P. aeruginosa*	Advanced disease; bronchiectatic change	TLR4, TLR5 (flagellin)	IL-8, IL-6, ROS	Biofilm, T3SS, quorum sensing	Pyocyanin generates ROS directly	Low–moderate (heavily confounded by disease severity)
Rhinovirus	Most frequent viral trigger	TLR3, RIG-I, MDA5	IFN-λ, IL-6, IL-8	Not persistent	Induces bacterial outgrowth (esp. *Haemophilus*); barrier disruption	Moderate–high (experimental infection data available)
Influenza	Severe epithelial injury	TLR3, TLR7, RIG-I	IL-6, TNF-α, type I IFN	Not persistent	Co-infection with NTHi aggravates lung injury and alters gut microbiota	Moderate
RSV	Increasingly recognised in adults	TLR4, TLR3, RIG-I	IL-6, IL-8, TSLP	Detectable in some stable patients	Possible chronic low-level inflammatory contribution	Low
SARS-CoV-2	Post-2020 addition	ACE2 entry; TLR7, TLR8	IL-6, TNF-α, complement	Not persistent (post-acute sequelae debated)	Endothelial injury and microvascular thrombosis; direct productive endothelial infection disputed	Moderate

LOS, lipooligosaccharide; T3SS, type III secretion system; ROS, reactive oxygen species. Evidence strength refers to the directness of the evidence for the proposed COPD-PH vascular pathway, as defined in the Literature Search and Review Approach section, and not to the methodological quality of the underlying studies. Cytokines listed are host-derived responses to microbial ligands.

**Table 3:** Airway and gut microbial alterations reported in COPD.

Compartment	Direction of Change	Specific Findings	Clinical Correlate
Airway—diversity	Reduced	Lower Shannon and Simpson indices; marked in *Haemophilus*-dominant patients	Associates with severity and exacerbation frequency
Airway—composition	Shift to *Proteobacteria*	*Haemophilus*, *Streptococcus*, *Moraxella* increased; *Prevotella*, *Veillonella* reduced	Present during stability, not only exacerbation
Airway—frequent exacerbators	Distinct signature	Fewer ASVs; distinct beta diversity; *Streptococcus*-dominant profiles	Associated with a history of frequent exacerbations (cross-sectional)
Airway—bacterial load	Increased	Correlates with IL-1β	Supports continuum rather than binary model
Gut—diversity	Reduced	Depletion of SCFA-producing taxa	Relates to lung function decline
Gut—metabolites	SCFAs reduced; TMAO increased	Impaired barrier integrity; LPS translocation; microbial TMA with hepatic conversion to TMAO	Linked to PH in experimental models

ASV, amplicon sequence variant; SCFA, short-chain fatty acid; TMA, trimethylamine; TMAO, trimethylamine N-oxide. The clinical correlates listed are associations reported in human COPD populations in which pulmonary vascular or haemodynamic endpoints were not measured; none of them constitutes direct evidence of a microbial contribution to pulmonary vascular remodelling in COPD-PH.

**Table 4:** Mediators linking microbial exposure to pulmonary vascular remodelling.

Mediator	Principal Source	Vascular Action	Evidence Type
IL-6	Macrophages, epithelium	PASMC proliferation; apoptosis resistance; overexpression sufficient to induce PH in animal models	Animal + human association
TNF-α	Macrophages, epithelium	eNOS impairment; adhesion molecule upregulation; endothelial apoptosis	Animal + human association
IL-1β	Inflammasome activation	Amplifies IL-6; correlates with sputum bacterial load	Human association
Endothelin-1	Endothelium (NF-κB-responsive)	Vasoconstriction via ETA; smooth muscle mitogenesis	Human + mechanistic
Nitric oxide (reduced)	Endothelium	Loss of vasodilation; eNOS uncoupling; ADMA inhibition	Human + mechanistic
Reactive oxygen species	Neutrophils, macrophages, pyocyanin	Endothelial injury; NO scavenging; redox signalling	Mechanistic
HIF-1α	Hypoxia and inflammation	Convergence point for hypoxic and inflammatory pathways	Animal + mechanistic
TGF-β	Multiple	Matrix deposition; EndoMT; smooth muscle proliferation	Animal + mechanistic
VEGF	Epithelium, endothelium	Endothelial maintenance; dysregulation in plexiform lesions	Mechanistic (bidirectional role)
Lipopolysaccharide	Gut translocation	CD14–MD-2–TLR4 activation (HMGB1 amplifying); increased endothelial permeability	Animal + human association
TMAO	Gut microbial TMA production; hepatic conversion to TMAO	HMGB1/TLR4 activation; endothelial permeability; correlates with PH severity	Human association
Short-chain fatty acids (reduced)	Gut microbial fermentation	Loss of HDAC inhibition; impaired endothelial barrier protection	Animal (interventional)

Evidence type denotes the directness of the evidence for the role of the mediator in COPD-PH, as defined in the Literature Search and Review Approach section. Human association refers to observational human data that have not been shown to be causal; animal and mechanistic entries have not been demonstrated to be active in human COPD-PH.

**Table 5:** Evidence for and against a causal contribution of microbial factors.

Domain	Supporting a Causal Role	Arguing for Caution	Evidence Tier for COPD-PH
Temporality	Dysbiotic signatures present during stability (cross-sectional); airway dysbiosis precedes lung-function decline	Vascular disease may itself predispose to colonisation	(2) Human COPD; no vascular endpoints
Independence from severity	Frequent-exacerbator phenotype stable across severity strata (ECLIPSE)	Severity confounds both exposure and outcome	(2) Human COPD; no vascular endpoints
Biological plausibility	Shared pathways: TLR→NF-κB→IL-6/TNF-α; ET-1 upregulation	Plausibility does not establish operation in vivo	(3) PAH/other PH; (4) animal and in vitro
Dose–response	Exacerbation frequency tracks lung function decline	Vascular dose–response not demonstrated	(2) Human COPD; no vascular endpoints
Experimental manipulation	Butyrate attenuates remodelling; microbiota modification suppresses PAH in models	Rodent hypoxic PH differs from human COPD-PH	(4) Animal
Compositional specificity	*Haemophilus*- and *Streptococcus*-dominant profiles associate with worse outcome	Could reflect airway conditions rather than cause them	(2) Human COPD; no vascular endpoints
Adjusted human data	—	No published cohort adjusts adequately for age, FEV_1_, hypoxaemia, comorbidity	(1) Direct human COPD-PH: none available

Evidence tier follows the hierarchy defined in the Literature Search and Review Approach section: (1) direct human COPD-PH evidence, in which pulmonary vascular or haemodynamic endpoints were measured; (2) human COPD evidence in which vascular endpoints were not measured; (3) evidence from pulmonary arterial hypertension or other pulmonary hypertension populations; and (4) animal and in vitro evidence. No row in this table rests on tier 1 evidence. Associations with exacerbation phenotype or with lung-function decline are tier 2 findings: they demonstrate neither temporality nor dose–response with respect to pulmonary vascular remodelling, and they should not be read as evidence that microbial factors cause pulmonary vascular remodelling in COPD-PH.

**Table 6:** Exacerbation-prevention interventions: evidence and caveats.

Intervention	Effect on Exacerbations	Evidence Quality (for the Effect in the Adjacent Column)	Vascular Outcome Data	Caveats
Influenza vaccination	Reduced total and influenza-related exacerbations	Low–moderate (Cochrane; few small RCTs; mortality underpowered)	None	Annual administration required
Pneumococcal vaccination	Reduced pneumonia and hospitalisation; effect varies with vaccine type, age and severity	Moderate	None	Serotype coverage varies; mortality benefit not established
RSV vaccination	Recently available for older adults	Emerging	None	COPD-specific data limited
Macrolide prophylaxis	RR 0.70 (95% CI 0.56–0.87); median time to first exacerbation 174→266 days	High (RCT + meta-analysis)	None	Hearing decrement 25% vs. 20%; resistance; QT prolongation
Pulmonary rehabilitation	Reduced hospitalisation; improved capacity	High	None	Access and adherence
Long-term oxygen therapy	Not an exacerbation-prevention intervention	High (haemodynamic endpoint)	Reduces PAP progression in severe hypoxaemia	Only intervention with demonstrated haemodynamic benefit
Smoking cessation	Reduced progression	High	Indirect	—
Probiotics/microbiome-directed	Under investigation	Low (pilot RCT)	None	Rationale from animal models

The evidence quality in this table is based on the framework defined in the Literature Search and Review Approach section: high indicates the evidence is randomised or otherwise direct human evidence for the effect listed in the adjacent column; moderate indicates the evidence is indirect or subgroup-dependent clinical evidence; low indicates the evidence is mechanistic or preclinical-support-only. Evidence is graded independently for each endpoint. The Evidence quality column refers only to the effect named in the adjacent column, which for every row except long-term oxygen therapy is an effect on exacerbations or on COPD progression; it does not refer to vascular outcomes. Vascular evidence is reported separately in the Vascular outcome data column: long-term oxygen therapy is the one listed intervention with demonstrated benefit on pulmonary haemodynamics in patients with severe resting hypoxaemia, whereas the remaining interventions have not been evaluated against pulmonary vascular endpoints. A reduction in exacerbations or in the rate of COPD progression should not be taken to imply an effect on pulmonary vascular remodelling.

**Table 7:** Vascular-directed therapy in COPD-PH: rationale versus evidence.

Agent Class	Physiological Rationale	Clinical Evidence	Guideline Position	Principal Risk
PDE-5 inhibitors (sildenafil, tadalafil)	Potentiates NO–cGMP; NO reduced in COPD-PH	Randomised acute study in COPD-PH: improved haemodynamics but reduced arterial oxygenation; no randomised evidence of clinical benefit; contrasting observational survival signal	No recommendation for COPD-PH; individualised specialist-centre decision in severe Group 3 PH; not recommended in non-severe PH-ILD (2022 ESC/ERS)	V′/Q′ mismatch, hypoxaemia
Endothelin receptor antagonists (bosentan, macitentan)	ET-1 elevated; blocks vasoconstriction and mitogenesis	Randomised trial of bosentan in severe COPD: no improvement in exercise capacity, with worsened hypoxaemia and quality of life; also unsuccessful in IPF	Not recommended	V′/Q′ mismatch; hepatotoxicity
Prostacyclin analogues (inhaled treprostinil)	Vasodilation; antiproliferative	PERFECT trial terminated early for increased serious adverse events	Not recommended	Harm signal
sGC stimulators (riociguat)	NO–cGMP pathway	RISE-IIP randomised trial in interstitial pneumonia PH terminated for excess deaths and SAEs; not studied in COPD-PH	Not recommended	Mortality signal in related population
Ensifentrine (PDE-3/4)	Bronchodilation plus anti-inflammatory	Highly preliminary: single-centre, retrospective, uncontrolled series of five patients; insufficient to establish efficacy	No position	Trials required
Long-term oxygen therapy	Reduces hypoxic pulmonary vasoconstriction (HPV)	Reduces PAP progression	Recommended in severe resting hypoxaemia	—

## Data Availability

No new data were created or analyzed in this study. Data sharing is not applicable to this article.

## Abbreviations

ADMA, asymmetric dimethylarginine; ASV, amplicon sequence variant; BAL, bronchoalveolar lavage; cGMP, cyclic guanosine monophosphate; COPD, chronic obstructive pulmonary disease; COPD-PH, COPD-associated pulmonary hypertension; CRP, C-reactive protein; eNOS, endothelial nitric oxide synthase; ERA, endothelin receptor antagonist; ET-1, endothelin-1; FEV_1_, forced expiratory volume in one second; HDAC, histone deacetylase; HIF, hypoxia-inducible factor; HMGB1, high mobility group box 1; IL, interleukin; LOS, lipooligosaccharide; LPS, lipopolysaccharide; mPAP, mean pulmonary arterial pressure; NF-κB, nuclear factor kappa B; NO, nitric oxide; NTHi, non-typeable *Haemophilus influenzae*; PASMC, pulmonary artery smooth muscle cell; PAWP, pulmonary artery wedge pressure; PDE, phosphodiesterase; PH, pulmonary hypertension; PVR, pulmonary vascular resistance; ROS, reactive oxygen species; RSV, respiratory syncytial virus; SCFA, short-chain fatty acid; TGF-β, transforming growth factor beta; TLR, Toll-like receptor; TMAO, trimethylamine N-oxide; TNF-α, tumour necrosis factor alpha; V′/Q′, ventilation–perfusion; VEGF, vascular endothelial growth factor; WU, Wood units.
